# Integration of single‐cell and bulk RNA‐sequencing data reveals the prognostic potential of epithelial gene markers for prostate cancer

**DOI:** 10.1002/1878-0261.13804

**Published:** 2025-02-19

**Authors:** Zhuofan Mou, Lorna W. Harries

**Affiliations:** ^1^ Department of Clinical and Biomedical Sciences, University of Exeter Medical School, Faculty of Health and Life Sciences University of Exeter UK

**Keywords:** bulk RNA‐seq, machine learning, prognosis, prostate cancer, single‐cell RNA‐seq, tumour microenvironment

## Abstract

Prognostic transcriptomic signatures for prostate cancer (PCa) often overlook the cellular origin of expression changes, an important consideration given the heterogeneity of the disorder. Current clinicopathological factors inadequately predict biochemical recurrence, a critical indicator guiding post‐treatment strategies following radical prostatectomy. To address this, we conducted a meta‐analysis of four large‐scale PCa datasets and found 33 previously reported PCa‐associated genes to be consistently up‐regulated in prostate tumours. By analysing single‐cell RNA‐sequencing data, we found these genes predominantly as markers in epithelial cells. Subsequently, we applied 97 advanced machine‐learning algorithms across five PCa cohorts and developed an 11‐gene epithelial expression signature. This signature robustly predicted biochemical recurrence‐free survival (BCRFS) and stratified patients into distinct risk categories, with high‐risk patients showing worse survival and altered immune cell populations. The signature outperformed traditional clinical parameters in larger cohorts and was overall superior to published PCa signatures for BCRFS. By analysing peripheral blood data, four of our signature genes showed potential as biomarkers for radiation response in patients with localised cancer and effectively stratified castration‐resistant patients for overall survival. In conclusion, this study developed a novel epithelial gene‐expression signature that enhanced BCRFS prediction and enabled effective risk stratification compared to existing clinical‐ and gene‐expression‐derived prognostic tools. Furthermore, a set of genes from the signature demonstrated potential utility in peripheral blood, a tissue amenable to minimally invasive sampling in a primary care setting, offering significant prognostic value for PCa patients without requiring a tumour biopsy.

## Graphical abstract

Prostate cancer is a leading malignancy with significant clinical heterogeneity in men. An 11‐gene signature derived from dysregulated epithelial cell markers effectively predicted biochemical recurrence‐free survival in patients who underwent radical surgery or radiotherapy. Post‐radiotherapy dysregulation of *FBP1*, *ERBB3*, *CACNA1D* and *APEX1* in localised patients also stratified overall survival in castration‐resistant cases using peripheral blood data. Created with BioRender.com.

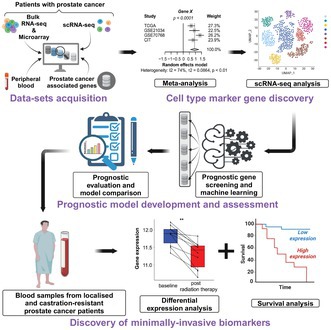

**Abbreviations**
AR: androgen receptor
BCR: biochemical recurrence
BCRFS: biochemical recurrence‐free survival
BH: Benjamini‐Hochberg
C‐index: concordance index
CRPC: castration‐resistant prostate cancer
EBRT: external beam radiation therapy
ECMG: epithelial cell marker gene
EMT: epithelial to mesenchymal transition
Enet: elastic net
EpCAM: epithelial cell adhesion molecule
FC: fold change
GBM: generalised boosted regression modelling
GDC: Genomic Data Commons
GEO: Gene Expression Omnibus
GO: Gene Ontology
GPS: Genomic Prostate Score
KEGG: Kyoto Encyclopedia of Genes and Genomes
KM: Kaplan–Meier
LASSO: least absolute shrinkage and selection operator
mCRPC: metastatic castration‐resistant prostate cancer
NCBI: National Center for Biotechnology Information
NK: natural killer
PBMC: peripheral blood mononuclear cell
PCa: prostate cancer
plsRcox: partial least squares regression for Cox
PRAD: prostate adenocarcinoma
PSA: prostate‐specific antigen
RNA: ribonucleic acid
RNA‐seq: RNA‐sequencing
ROC: receiver operating characteristic
RP: radical prostatectomy
RSF: random survival forest
scRNA‐seq: single‐cell RNA‐sequencing
SuperPC: supervised principal components
SVM: support vector machine
TCGA: The Cancer Genome Atlas
UMAP: uniform manifold approximation and projection

## 1 Introduction

Prostate cancer (PCa) is a major global health concern for men and a leading cause of cancer‐related deaths, highlighting the urgent need for enhanced diagnostics and treatments [1, 2]. The lack of symptoms in the early stages of PCa makes it difficult to detect. Conventional diagnostic methods, such as serum prostate‐specific antigen (PSA) testing, digital rectal examinations and imaging methods such as ultrasound and magnetic resonance imaging, have limitations and do not always provide accurate early detection. The insufficiency of these approaches is particularly evident in patients who are diagnosed with PCa and experience biochemical recurrence (BCR) and re‐evaluation after treatment [3].

The PSA test remains the primary screening tool for PCa, but its high false positive rate has made it controversial. This can lead to over‐diagnosis and unnecessary treatment, increasing patient anxiety and the risk of complications from treatments that may not have been needed [4, 5, 6]. The inherent heterogeneity of localised PCa presents a wide range of clinical outcomes. Radical prostatectomy (RP), a surgical removal of the prostate and external beam radiation therapy (EBRT) are primary treatments for localised early‐stage PCa and usually result in favourable oncologic outcomes [7, 8]. However, ~ 20–40% of patients will experience BCR, an increase in PSA levels, after RP treatment, which can progress to advanced PCa [9, 10, 11, 12]. Similarly, 22–69% of patients undergoing EBRT treatment can experience BCR [7, 13]. This highlights the critical need for developing biomarkers that can accurately predict BCR at an early or localised stage and therefore enhancing PCa management.

Recent advancements in computational methods and genomic research have enabled more accurate biomarker discovery, with meta‐analyses playing a crucial role in identifying consistent expression changes across independent datasets. A variety of tools have been developed to simplify the meta‐analysis pipeline and enhance its accuracy in identifying robust biomarkers for PCa [14]. For decades, the integration of biomarkers and gene expression signatures derived from microarray and RNA‐seq data has played an important role in advancing the risk assessment of PCa patients. These technologies have substantially improved clinicians' ability to predict clinical outcomes and making therapeutic decisions, including the determination of active surveillance needs and the customisation of treatment intensity. Commercial gene signature tests such as Oncotype Dx Genomic Prostate Score (GPS) [15, 16], Prolaris [17] and Decipher [18] have now been used in clinical practice for PCa, demonstrating the practical values and applicability of these analytical approaches.

One noteworthy observation is that these signatures rarely take into consideration the cell origin of the signals used to define the signatures. The tumour microenvironment plays a critical role in the development, growth, progression, metastasis and response to the treatment of PCa [19]. Expression changes may have different biological consequences in different cell types within a tissue, and biologically relevant changes in expression in a small subset of clinically relevant cell types often goes undetected in bulk sequencing analyses. Analysis of effects at the level of single cells provides detailed insights into the tumour microenvironment by elucidating the interactions among different cell types, including cancer cells, stromal cells, immune cells and the extracellular matrix [20], and is arguably a better choice when dealing with a complex condition with considerable heterogeneity such as PCa. An integrative approach that utilises both bulk RNA‐seq and scRNA‐seq data allows for a more comprehensive assessment of gene expression on a cellular level, revealing potential diagnostic and prognostic biomarkers in cancer [21, 22, 23, 24]. Full clinical utility is also limited by the need to access tumour tissue for current signatures. A biomarker set that translates from the site of the tumour into a tissue such as peripheral blood that can be accessed in a minimally invasive manner raises the possibility of widening accessibility and utility in a primary care setting.

In this study, we assessed the expression changes of 48 previously identified PCa‐associated genes across a range of bulk RNA‐seq datasets. Genes showing concordant responses across the datasets were then queried at single‐cell resolution in a single‐cell RNA‐sequencing (scRNA‐seq) dataset. We then applied advanced machine learning algorithms to develop a prognostic signature based on cell type‐specific significant genes, which showed robust predictive accuracy for survival outcomes in localised PCa patients. Finally, we queried the expression of signature genes in 2 peripheral blood datasets to assess translation potential to a minimally invasive tissue; a necessary prerequisite for monitoring treatment and predicting overall survival in PCa patients. This comprehensive approach seeks to enhance prognostic precision and inform better clinical management of PCa.

## 2 Materials and methods

### 2.1 Data acquisition and preprocessing of prostate cancer cohorts

Primary PCa and blood‐based transcriptomic datasets were systematically retrieved from established public databases, including the Genomic Data Commons (GDC) [25], cBioPortal [26], the National Center for Biotechnology Information (NCBI) Gene Expression Omnibus (GEO) [27], ArrayExpress [28] and PCaDB [29].

To facilitate a comprehensive analysis of expression changes within PCa tumours and assess biochemical recurrence‐free survival (BCRFS), five large‐scale independent primary PCa cohorts were selected: TCGA‐PRAD (*n* = 547), GSE21034 (*n* = 160), GSE70768 (*n* = 199), E‐MTAB‐6128 (*n* = 141) and DKFZ (*n* = 118). The selection of these cohorts was based upon two criteria: a minimum sample size exceeding 100, and the availability of comparative data for primary tumours against normal prostate samples, or BCRFS data. The datasets, profiled through either microarray or bulk RNA sequencing, underwent rigorous preprocessing, detailed in a previous study [29], and were accessible via the public database PCaDB (http://bioinfo.jialab‐ucr.org/PCaDB/; accessed on 02/10/2023). Clinical data, including Gleason scores, clinicopathological factors and BCRFS information, were obtained from PCaDB for each cohort. In addition, the Belfast radiotherapy cohort (GSE116918) served to assess the prognostic value of the signature risk score in determining PCa treatment response.

The scRNA‐seq dataset (GSE193337; 10x Genomics), derived from prostate tumour tissues of four patients with localised PCa after RP, was downloaded from GEO and used to identify cell‐specific marker genes.

Lastly, two blood‐based transcriptomic datasets targeting gene expression analysis in PCa patients were obtained from GEO. These included GSE30174, featuring samples from non‐metastatic PCa patients 4 weeks after EBRT and their matched controls, along with GSE53922, containing peripheral blood mononuclear cell (PBMC) samples from castration‐resistant prostate cancer (CRPC) patients, inclusive of overall survival data. A summary of all datasets used in this study is provided in Table 1.

**Table 1:** Overview of the cohorts used. ‘/’, not available; BCR, biochemical recurrence; OS, overall survival; PRAD, prostate adenocarcinoma; TCGA, The Cancer Genome Atlas.

Dataset name	Title	GEO/ArrayExpress accession	Total number of samples	Availability of survival endpoint	Platform	Technology	References
TCGA‐PRAD	Prostate Adenocarcinoma (The Cancer Genome Atlas)	/	547 (52 normal vs. 495 primary tumour)	BCR	Illumina HiSeq 2000	RNA sequencing	[25]
Taylor/MSKCC	Whole‐transcript and exon‐level expression data for human primary and metastatic prostate cancer samples and control normal adjacent benign prostate	GSE21034	160 (29 normal vs. 131 primary tumour)	BCR	Affymetrix Human Exon 1.0 ST Array	Microarray	[80]
Cambridge	Prostate cancer stratification using molecular profiles [CamCap ExpressionArray]	GSE70768	199 (74 normal vs. 125 primay tumour)	BCR	Illumina HumanHT‐12 V4.0 Expression Beadchip	Microarray	[81]
CIT	Expression array for Multi‐omics molecular profiling of primary prostate adenocarcinoma	E‐MTAB‐6128	141 (40 normal vs. 101 primary tumour)	BCR	Affymetrix Human Gene 2.0 ST Array	Microarray	[82]
DKFZ	Prostate Cancer (DKFZ, Cancer Cell 2018)	/	118 (tumour only)	BCR	Illumina HiSeq 2000	RNA sequencing	[83]
Belfast	Using biopsies to improve risk stratification in patients with prostate cancer treated with radical radiation therapy	GSE116918	248 (tumour only)	BCR Metastasis	Almac Diagnostics Prostate Disease Specific Array (DSA)	Microarray	[84]
GSE193337	Single‐cell and bulk RNA‐seq of human tumour‐benign prostate adenocarinoma samples	GSE193337	4 (tumour only)	/	Illumina HiSeq 4000 (Homo sapiens)	Single‐cell RNA sequencing	[85]
GSE30174	Molecular‐genetic correlates of fatigue in cancer patients receiving localised external beam radiation therapy	GSE30174	20 (10 case vs 10 matched control)	/	Affymetrix Human Genome U133 Plus 2.0 Array	Microarray	[86]
GSE53922	Gene expression profiles of PBMC in CRPC patients receiving persornalized peptide vaccination	GSE53922	112 PBMC samples	OS	Illumina HumanWG‐6 v3.0 expression beadchip	Microarray	[87]

### 2.2 Gene panel and meta‐analysis

A meta‐analysis of the expression patterns of 48 previously implicated PCa‐associated genes [30, 31] was carried out across four independent PCa cohorts: TCGA‐PRAD, GSE21034, GSE70768 and E‐MTAB‐6128. For genes mapped to multiple symbols or Entrez IDs, the one with the highest mean expression was selected for downstream analysis. Using the R package *GeneMeta*, we estimated the effect size of each gene within‐dataset as the log2 transformed fold change, using Hedges' *g* to determine the standardised mean difference, following the recommendations [32]. These effect sizes were then combined through an inverse‐variance random‐effects model [33], calculating both the pooled effect size and its standard error per gene. Genes were considered significantly dysregulated if they had a Benjamini‐Hochberg (BH) adjusted *P*‐value < 0.05. For each significant gene, a meta forest plot was generated using the R package *meta* to visually summarise the findings [34].

### 2.3 Single‐cell RNA‐sequencing analysis and identification of marker genes

To identify cell type specific marker genes, the scRNA‐seq dataset GSE193337 was analysed, comprising four primary prostate tissue samples obtained from patients after RP. Raw data processed by Cell Ranger were acquired from the GEO and imported into R for further analysis. A Seurat object was created for each sample using the R package *Seurat* [35, 36], focusing on cells where at least 100 genes were detected. Quality control criteria included the selection of cells expressing over 200 genes but with fewer than 20 000 total counts, ensuring less than 20% of the reads were mitochondrial, to mitigate the impact of degraded or apoptotic cells.

For the purpose of dimensionality reduction, 2000 genes demonstrating the most significant variance across the PCa samples were chosen for principal component analysis. The first 20 principal components were used to visualise cell clusters with uniform manifold approximation and projection (UMAP). Sample integration was conducted using canonical correlation analysis, with batch effects from patient‐specific tumour samples corrected using the function *FindintegrationAnchors*. Cell clusters were identified using a resolution level of 0.6 and were annotated with major cell types using the automatic annotation tool *SingleR* [37], based on the Human Primary Cell Atlas reference dataset [38]. To identify cell type specific marker genes, we applied the function *FindAllMarkers* to the tumour samples, together with the Wilcoxon Rank Sum test with thresholds of |log_2_ fold change (log_2_FC)| > 0.1 and a BH adjusted *P*‐value < 0.05.

### 2.4 GO and KEGG pathway analysis

To investigate the biological relevance of the identified cell marker genes that also show to be consistently dysregulated in PCa tumours from the bulk analysis, we conducted Gene Ontology (GO) [39] and Kyoto Encyclopedia of Genes and Genomes (KEGG) pathway analysis [40] using the R package *clusterprofiler* [41, 42]. GO terms and KEGG pathways are considered to be significant if BH adjusted *P*‐value < 0.05.

### 2.5 Development and validation of cell type‐specific prognostic signature using advanced machine learning methods

Our scRNA‐seq analysis indicated that the majority of consistently dysregulated genes were enriched in the epithelial cells. We therefore assessed these genes for their potential as cell type‐specific prognostic candidates to predict BCRFS in PCa patients. Gene expression data were standardised across datasets using z‐score transformation to ensure consistent scaling and variance, enhancing the efficacy of the subsequent machine learning algorithmic comparisons and learning processes. For model training and testing, the five PCa cohorts (i.e., TCGA‐PRAD, GSE21034, GSE70768, E‐MTAB‐6128 and DKFZ) were used and the clinical characteristics for each are summarised in Table 2.

**Table 2:** Clinical characteristics of five cohorts from primary prostate tumours in prostate cancer patients. Age, age at diagnosis; BCRFS, biochemical recurrence‐free survival; N stage, lymph node status (N0 = without lymph node metastasis; N1, with lymph node metastasis); PRAD, prostate adenocarcinoma; PSA, prostate‐specific antigen; T stage, tumour stage; TCGA, The Cancer Genome Atlas.

Clinical feature	TCGA‐PRAD (*n* = 382)	GSE21034 (Taylor) (*n* = 130)	GSE70768 (Cambridge) (*n* = 90)	E‐MTAB‐6128 (CIT) (*n* = 75)	DKFZ (*n* = 105)
Tissue	Prostate	Prostate	Prostate	Prostate	Prostate
Age (year) (%)					
< 60	150 (39.3%)	/	38 (42.2%)	18 (24%)	105 (100%)
≥ 60	232 (60.7%)	/	52 (57.8%)	57 (76%)	/
Pathological T stage (%)					
T1‐2	130 (34.0%)	/	24 (26.7%)	/	68 (64.8%)
T3‐4	252 (66.0%)	/	66 (73.3%)	/	37 (35.2%)
Pathological N stage (%)					
N0	310 (81.2%)	/	82 (91.1%)	/	/
N1	72 (18.8%)	/	8 (8.9%)	/	/
Gleason score (%)					
≤ 7	207 (54.2%)	115 (88.5%)	84 (93.3%)	64 (85.3%)	91 (86.7%)
> 7	175 (45.8%)	15 (11.5%)	6 (6.7%)	11 (14.7%)	14 (13.3%)
PSA (ng·mL^−1^) (%)					
≤ 10	252 (66.0%)	/	65 (72.2%)	63 (84%)	58 (55.2%)
> 10	130 (34.0%)	/	25 (27.8%)	12 (16%)	47 (44.8%)
Survival type	BCRFS	BCRFS	BCRFS	BCRFS	BCRFS
Number of events (%)	90 (23.6%)	27 (20.8%)	15 (16.7%)	12 (16%)	24 (22.9%)

We integrated a total of 97 algorithmic combinations from 10 machine learning models, including elastic net (Enet), least absolute shrinkage and selection operator (LASSO), Ridge, stepwise Cox, CoxBoost, partial least squares regression for Cox (plsRcox), survival support vector machine (survival‐SVM), supervised principal components (SuperPC), generalised boosted regression modelling (GBM) and random survival forest (RSF). These models were fitted using a 10‐fold cross‐validation approach, trained and tested initially in the TCGA‐PRAD and then tested in external GSE21034, GSE70768, E‐MTAB‐6128 and DKFZ cohorts. The implementation of these models used specific R packages refined to each algorithm: the Enet, LASSO and Ridge analyses were conducted using the package *glmnet*, RSF analysis was performed with the package *randomForestSRC* and the stepwise Cox model, CoxBoost, plsRcox, SuperPC, GBM and survival‐SVM were implemented via their respective packages: *survival*, *CoxBoost*, *plsRcox*, *superpc*, *gbm* and *survivalsvm*. The performance of each model was evaluated using Harrell's concordance index (C‐index) as a metric for predictive accuracy, with the optimal model selected based on the highest average C‐index across the five cohorts.

The resulting epithelial‐based multi‐gene signature's effectiveness in prognosis was initially assessed through Kaplan–Meier (KM) survival analysis with log‐rank test, classifying patients into high‐risk and low‐risk groups. More specifically, patients in our study were stratified into high‐ and low‐risk groups based on either gene expression or risk scores using the surv_cutpoint function from the R package *survminer*, which utilises the maximally selected rank statistics (maxstat) method to determine the optimal cut point. Additionally, the signature's accuracy in predicting survival years was examined and visualised through the area under the curve of time‐dependent receiver operating characteristic (ROC) analysis using the R package *timeROC* [43].

### 2.6 Comparison with published signatures

To assess the effectiveness of the developed epithelial signature in predicting BCRFS for patients diagnosed with PCa, we searched and carefully selected published gene signatures from the PubMed. In total, 68 gene signatures were considered, including 30 high‐quality PCa prognostic signatures identified in a previous study [29]. This selection also included commercially available signatures such as Decipher, Prolaris and GPS. The predictive accuracy of each signature's risk score was evaluated using the C‐index, providing a comprehensive comparison of their ability to predict BCRFS in PCa patients across the studied cohorts.

### 2.7 Tumour microenvironment of the epithelial signature

Tumour samples from the TCGA‐PRAD dataset were analysed using the R package *ESTIMATE* [44] to assess the distribution of immune and stromal cells within the tumour microenvironment across patient groups categorised by their risk level according to the epithelial cell marker gene (ECMG)‐based signature. This analysis produces TME scores, which serve to quantify scores that positively correlate with proportions of immune (ImmuneScore), stromal (StromalScore) and overall (ESTIMATEScore) components, inferred from the epithelial signature risk scores.

The R package *CIBERSORT* [45] was applied to measure the relative abundances of 22 immune cell types for each PCa tumour sample based on the expression data. Then for each immune cell type, the difference was compared between the high‐ and low‐risk patient groups identified by the epithelial signature risk scores.

Wilcoxon test was used to compare between the two risk groups, with *P* < 0.05 as significance threshold. Non‐parametric Spearman correlation analysis was used for the associated between the epithelial signature risk score and relative abundance of each immune cell. *P*‐value < 0.05 was used as the significance threshold.

### 2.8 Investigation of potential biomarkers from blood samples

Leveraging the advantages of liquid biopsies as minimally invasive and easily accessible sources of test material that might be amenable to routine clinical evaluation, we used two blood‐based transcriptomic datasets. Herein, we used dataset GSE30174, which features white blood cells isolated from 10 non‐metastatic PCa patients 4 weeks post‐EBRT, alongside matched baseline and healthy samples. Moreover, dataset GSE53922 was analysed, comprising PBMC samples from 112 PCa patients, annotated with overall survival data. These analyses aimed to investigate the clinical applicability of identified epithelial signature genes as potential biomarkers in diagnosing and monitoring PCa progression, as well as prognosing of patients' overall survival. The differential expression was assessed using the paired non‐parametric Wilcoxon signed‐rank test, with a significance threshold set at *P* < 0.05. The KM analysis and log‐rank test (significance threshold: *P* < 0.05) were used for the association between the optimally classified patients in association with their overall survival.

## 3 Results

### 3.1 Meta‐analysis identifies consistently dysregulated genes in PCa

**Fig. 1:**
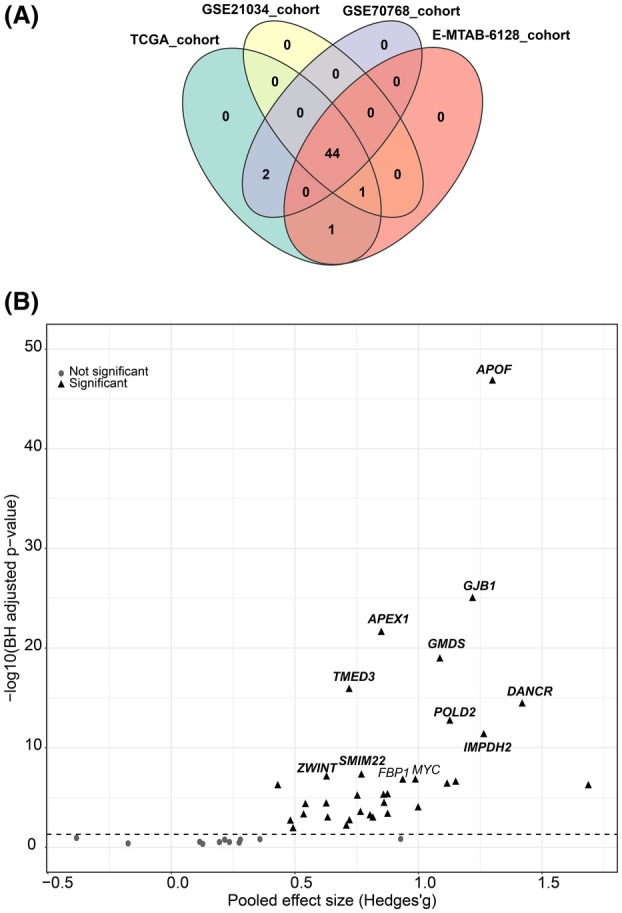
Meta‐analysis and screening for prostate cancer associated genes across four large‐scale cohorts (TCGA‐PRAD, GSE21034, GSE70768, and E‐MTAB‐6128). (A) A Venn diagram illustrating the overlap of detected genes among the four cohorts. (B) A volcano plot highlighting consistently dysregulated genes (represented by triangles) and stable genes (represented by circles) across the cohorts. The *x*‐axis represents the pooled effect size (Hedges' *g*) and the *y*‐axis denotes the Benjamini‐Hochberg (BH) adjusted *P*‐value, transformed using the negative logarithm to base 10. The horizontal dashed line indicates the threshold for the BH adjusted *P*‐value of 0.05. TCGA‐PRAD: The Cancer Genome Atlas Prostate Adenocarcinoma.

Of the 48 genes assessed, 44 were detected to be expressed in all four independent PCa datasets (TCGA‐PRAD, GSE21034, GSE70768, E‐MTAB‐6128) (Fig. 1A). Subsequent meta‐analysis identified 33 genes significantly upregulated in PCa tumours (Fig. 1B and Table S1). The top genes identified were *APOF* (pooled Hedges' *g* = 1.299, *P* < 0.001), *GJB1* (pooled Hedges' *g* = 1.219, *P* < 0.001), *APEX1* (pooled Hedges' *g* = 0.850, *P* < 0.001), *GMDS* (pooled Hedges' *g* = 1.087, *P* < 0.001), *TMED3* (pooled Hedges' *g* = 0.720, *P* < 0.001) and *DANCR* (pooled Hedges' *g* = 1.420, *P* < 0.001). These upregulated genes are individually visualised in Fig. S1.

### 3.2 Identification of ECMGs using scRNA‐seq cohorts

**Fig. 2:**
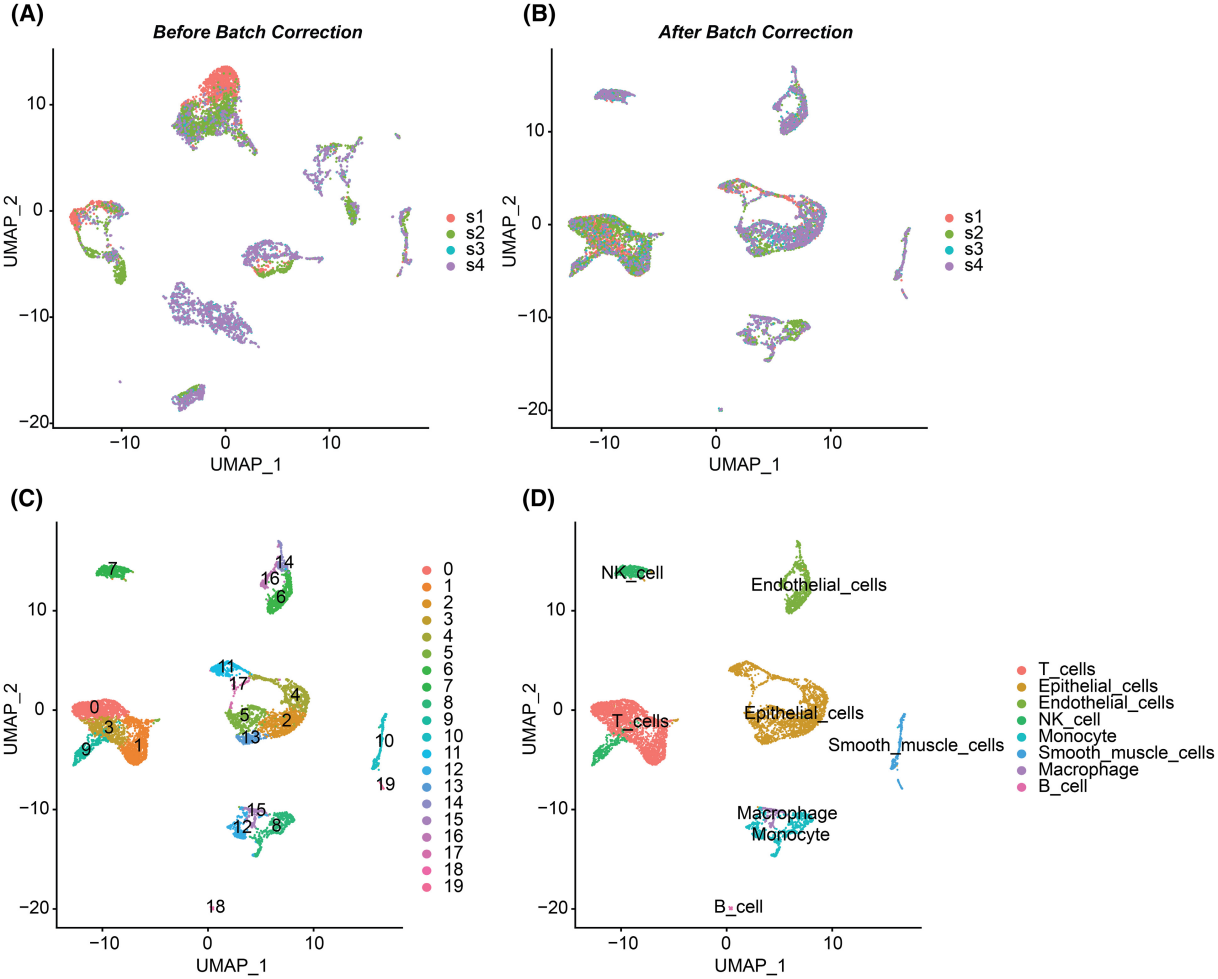
Single‐cell RNA sequencing analysis of dataset GSE193337. (A) Uniform manifold approximation and projection (UMAP) clustering showing the distribution of cells from four tumour samples before batch correction. (B) UMAP clustering showing the distribution of cells from the four tumour samples after batch correction. (C) UMAP clustering showing the distribution of identified cell clusters. (D) UMAP clusters annotated by their respective cell types. NK, natural killer.

**Fig. 3:**
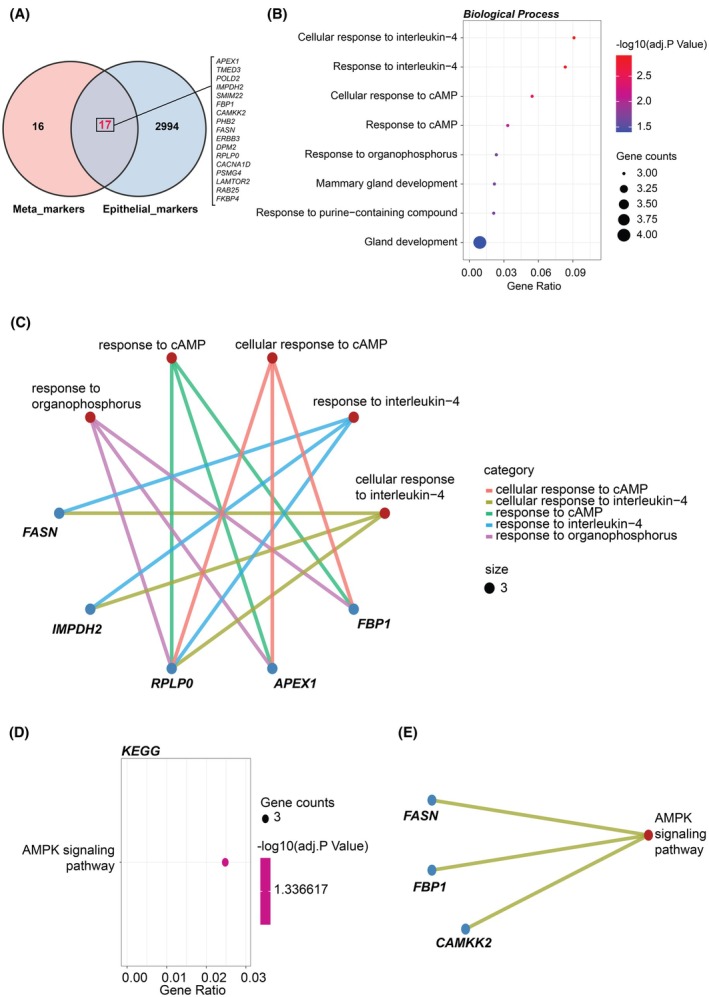
Enrichment analysis of the 17 dysregulated PCa‐associated epithelial cell marker genes. (A) A Venn diagram illustrating the intersection between epithelial cell marker genes and the 33 genes identified as differentially expressed in prostate cancer through meta‐analysis. (B) Enriched Gene Ontology (GO) terms for biological processes associated with these genes. (C) The specific genes corresponding to each GO term. (D) Enriched Kyoto Encyclopedia of Genes and Genomes (KEGG) pathways. (E) The specific genes corresponding to each KEGG pathway.

The scRNA‐seq dataset GSE193337 was subjected to a principal component analysis focusing on the top 2000 variable genes across four PCa samples. The first 20 principal components were considered for UMAP clustering and visualisation. UMAP clustering of cells from the four PCa tumours, before and after batch correction, is presented in Fig. 2A,B, respectively. We identified a total of 20 distinct cell clusters (Fig. 2C) and categorised them into eight major cell types (Fig. 2D). By overlapping dysregulated PCa‐associated genes with marker genes from various cell types, we found that more than half (17 out of 33) were derived from epithelial cells (Table S2 and Fig. 3A). Additionally, we identified marker genes in other cell types: 11 in T cells, 8 in smooth muscle cells, 4 in macrophages, 3 in endothelial cells, 3 in natural killer (NK) cells and 3 in monocytes, with none in B cells (Fig. S2). The single‐cell expression profiles of the 17 ECMGs are presented in Fig. S3 and were used for further downstream analyses.

### 3.3 Enrichment analysis of PCa‐associated ECMGs

GO and KEGG analyses showed that the identified 17 dysregulated ECMGs were significantly enriched in some cellular responses, including ‘Cellular response to interleukin‐4’ (with enriched genes *FASN*, *IMPDH2* and *RPLP0*) and ‘Cellular response to cAMP’ (with enriched genes *RPLP0*, *APEX1* and *FBP1*), as well as ‘Gland development’ (Fig. 3B,C). KEGG pathway analysis showed these were also enriched in ‘AMPK signalling pathway’, with enriched genes *FASN*, *FBP1* and *CAMKK2* (Fig. 3D,E).

### 3.4 Establishment of ECMG‐based signature using RSF model

**Fig. 4:**
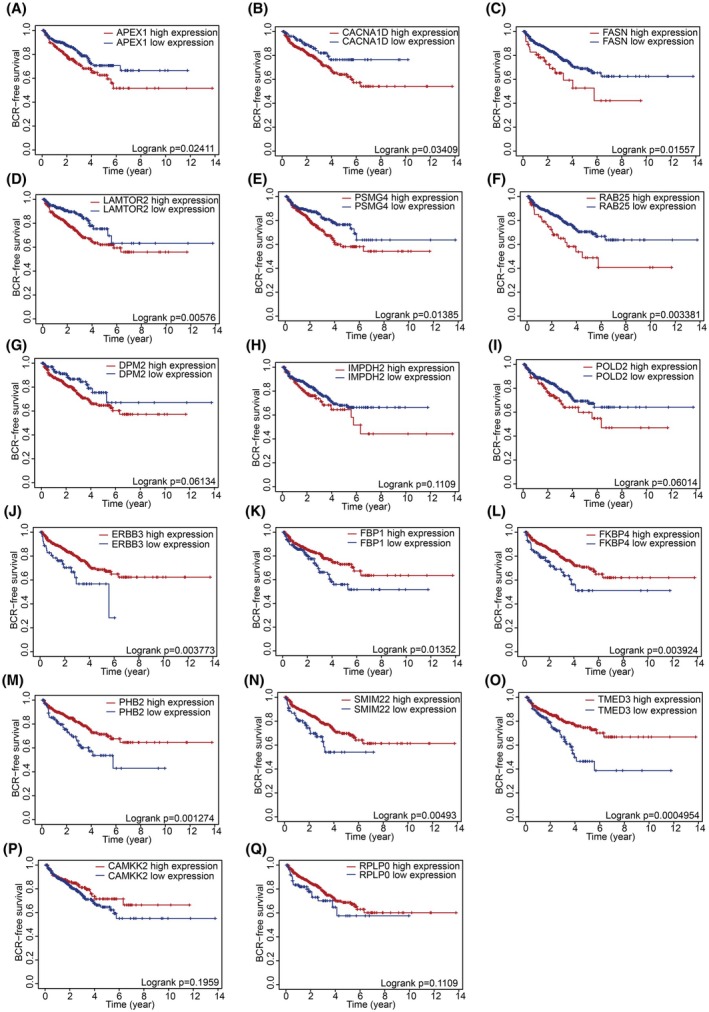
Kaplan–Meier survival analysis of candidate epithelial marker genes in prostate cancer. (A–Q) Kaplan–Meier survival curves complemented by log‐rank tests for each of the overlapping genes associated with prostate cancer, demonstrating their potential prognostic significance.

To identify a prognostic signature based on the expression of 17 PCa‐associated ECMGs, we initially evaluated their prognostic relevance to BCRFS in PCa patients using KM survival analysis. This revealed that 12 of these genes had significant prognostic value (Table S3). Specifically, patients with high expression of *APEX1*, *CACNA1D*, *FASN*, *LAMTOR2*, *PSMG4* and *RAB25* had significantly worse BCRFS compared to those with low expression (Fig. 4A–F; respectively). In contrast, high expression of *ERBB3*, *FBP1*, *FKBP4*, *PHB2*, *SMIM22* and *TMED3* was associated with a significantly improved BCRFS compared to their low expression counterparts (Fig. 4J–O; respectively). Genes that were not significant are those shown in Fig. 4G–I,P,Q.

Following the evaluation of 97 machine learning algorithms, trained on the TCGA‐PRAD dataset and tested across GSE21034, GSE70768, E‐MTAB‐6128 and DKFZ cohorts, the RSF model demonstrated the highest mean C‐index at 0.749 (Fig. S4A and Table S4). Among the 11 genes assessed and included in the RSF model, *LAMTOR2*, *RAB25*, *FBP1*, *TMED3* and *FASN* emerged as the top five based on their relative importance scores, each exceeding the threshold of 0.4 (Fig. S4B). Given its superior performance, the RSF model was selected as the optimal model for further evaluation. A summary of the 11 genes in relation to prostate cancer is shown in Table S5.

### 3.5 Prognostic evaluation and comparison of ECMG‐based signature with published PCa signatures

**Fig. 5:**
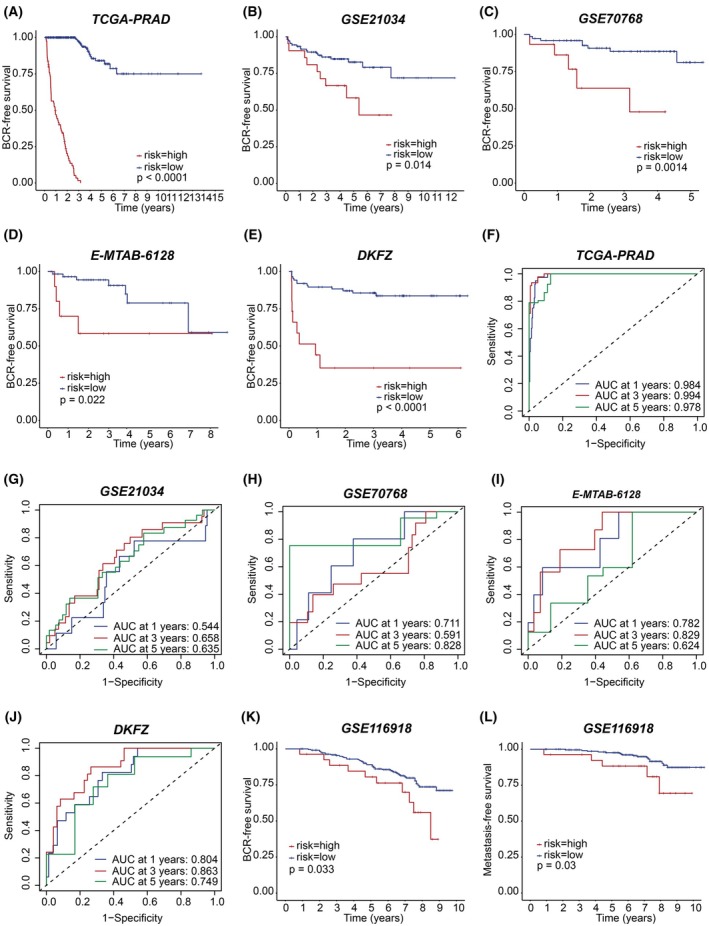
Evaluation of the epithelial cell marker gene based signature for prognosis of patients diagnosed with prostate cancer. Kaplan–Meier (KM) survival analysis with log‐rank test for biochemical recurrence‐free survival (BCRFS) in TCGA‐PRAD (A), GSE21034 (B), GSE70768 (C), E‐MTAB‐6128 (D), and DKFZ (E). Receiver operating characteristic analysis for BCRFS in TCGA‐PRAD (F), GSE21034 (G), GSE70768 (H), E‐MTAB‐6128 (I), and DKFZ (J). KM survival analysis with log‐rank test in GSE116918 for BCRFS (K) and for metastasis‐free survival (L). BCR, biochemical recurrence; PRAD, prostate adenocarcinoma; TCGA, The Cancer Genome Atlas.

The ECMG‐based signature could effectively classify patients into high‐risk and low‐risk categories, with significant differences in BCRFS observed across all five PCa cohorts. Specifically, high‐risk patients exhibited poorer BCRFS outcomes compared to low‐risk patients, as evidenced by log‐rank *P*‐values (TCGA‐PRAD: *P* < 0.0001; GSE21034: *P* = 0.014; GSE70768: *P* = 0.0014; E‐MTAB‐6128: *P* = 0.022; DKFZ: *P* < 0.0001; Fig. 5A–E, respectively). ROC analysis demonstrated robust predictive capability of the signature for 1‐, 3‐ and 5‐year BCRFS in PCa patients across the five cohorts (TCGA‐PRAD: 0.984, 0.994, 0.978; GSE21034: 0.544, 0.658, 0.635; GSE70768: 0.711, 0.591, 0.828; E‐MTAB‐6128: 0.782, 0.829, 0.624; DKFZ: 0.804, 0.863, 0.749 for 1‐, 3‐ and 5‐year BCRFS, respectively; Fig. 5F–J, respectively). Moreover, this signature could also differentiate patients following radical radiotherapy, with high‐risk patients exhibiting worse BCRFS and metastasis‐free survival compared to their low‐risk counterparts (Fig. 5K,L, respectively).

We also observed a higher frequency of gene mutations in patients from the high‐risk group compared to those in the low‐risk group. Among the top mutated genes, *SPOP* and *TP53* were the most frequently mutated in the high‐risk group, occurring in 16% and 17% of the samples, respectively (Fig. S5A). Conversely, the gene *TTN* exhibited the highest mutation frequency in the low‐risk group, occurring in 11% of the samples (Fig. S5B).

**Fig. 6:**
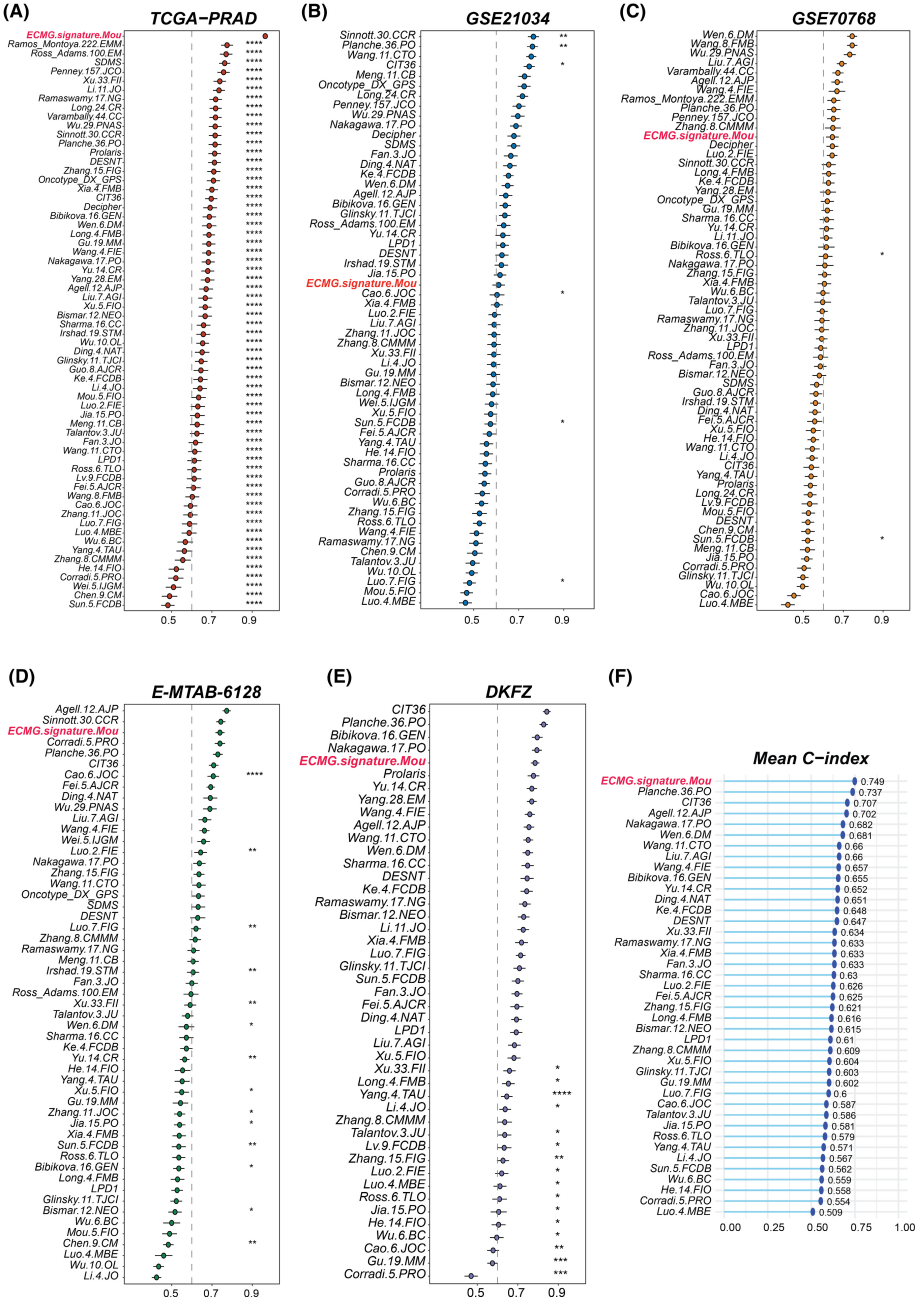
Comparative analysis of epithelial cell marker gene based signature against 68 published signatures. Concordance index (C‐index) values for each signature were calculated and compared to assess their predictive power in (A) TCGA‐PRAD, (B) GSE21034, (C) GSE70768, (D) E‐MTAB‐6128 and (E) DKFZ cohorts. (F) Displays the average C‐index for each signature across the five cohorts, with subsequent ranking to highlight the relative performance. PRAD, prostate adenocarcinoma; TCGA, The Cancer Genome Atlas.

Moreover, in the comparative analysis with published PCa gene signatures, our ECMG‐based signature demonstrated superior predictive performance. Specifically, it ranked first among all evaluated signatures in the TCGA‐PRAD cohort, showing its robust prognostic value (Fig. 6A). In the GSE21034 cohort, it achieved a middle‐ranking position (Fig. 6B). Our signature ranked 12th in the GSE70768 cohort (Fig. 6C), third in the E‐MTAB‐6128 cohort (Fig. 6D) and fifth in the DKFZ cohort (Fig. 6E), reflecting its varied yet significant prognostic utility across different datasets. Notably, when considering the overall performance across all cohorts, our ECMG‐based signature outperformed all compared signatures, including those commercial ones, suggesting its effectiveness in prognostic prediction of BCRFS for PCa patients (Fig. 6F).

### 3.6 Clinical significance of ECMG‐based signature

Comparing the C‐index against conventional clinicopathological parameters, the ECMG signature demonstrated the best prognostic performance in predicting BCRFS, except in the E‐MTAB‐6128 and GSE21034 datasets, where the Gleason score exhibited a marginally better C‐index than the ECMG signature (Fig. S6A–E). Univariate Cox regression analysis revealed the ECMG signature as a significant risk factor (*P* < 0.05 and hazard ratio (HR) > 1) for BCRFS prognosis in the three larger cohorts (TCGA‐PRAD, GSE21034 and DKFZ; Tables 3, 4, 5, respectively), while *P*‐values were slightly above 0.05 in the two smaller cohorts (GSE70768 and E‐MTAB‐6128; Tables 6 and 7, respectively). Furthermore, multivariate Cox regression analysis suggested its potential utility as an independent clinical prognostic parameter over conventional variables in the three larger cohorts (*P* < 0.05; Tables 3, 4, 5, respectively). Moreover, we found that PCa patients with higher age, pathological T and N stage, PSA and Gleason score also had significantly higher risk scores in the two largest cohorts TCGA‐PRAD (Fig. S7A) and DKFZ (Fig. S7B). These findings suggest the clinical significance of our ECMG signature, particularly in larger cohorts.

**Table 3:** Results of univariate and multivariate Cox regression analyses for conventional clinicopathological parameter and ECMG signature risk score in TCGA‐PRAD cohort. Age, age at diagnosis; ECMG, epithelial cell marker gene; N stage, lymph node status (N0 = without lymph node metastasis; N1 = with lymph node metastasis); PRAD, prostate adenocarcinoma; PSA, prostate‐specific antigen; T stage, tumour stage; TCGA, The Cancer Genome Atlas.

Univariate cox regression					Multivariate cox regression				
Id	HR	HR.95 L	HR.95H	*P* value	Id	HR	HR.95 L	HR.95H	*P* value
Age	1.188969	0.775972	1.821775	0.426618512	Age	0.89428	0.550568	1.452566	0.651642
Pathological T stage	4.665947	2.415126	9.014462	4.56E‐06	Pathological T stage	1.056739	0.513497	2.174692	0.880862
Pathological N stage	2.333913	1.485322	3.667321	0.000237082	Pathological N stage	1.398699	0.846108	2.312188	0.190749
PSA	1.36281	0.895345	2.074342	0.148680883	PSA	1.756587	1.100729	2.803231	0.018157
Gleason score	4.04442	2.532374	6.459287	4.92E‐09	Gleason score	0.898401	0.529905	1.523149	0.690802
ECMG risk score	1.185202	1.159291	1.211692	2.71E‐51	ECMG risk score	1.196067	1.166621	1.226257	5.24E‐45

**Table 4:** Results of univariate and multivariate Cox regression analyses for conventional clinicopathological parameter and ECMG signature risk score in GSE21034 cohort. ECMG, epithelial cell marker gene.

Univariate Cox regression					Multivariate Cox regression				
Id	HR	HR.95 L	HR.95H	*P* value	Id	HR	HR.95 L	HR.95H	*P* value
Gleason score	10.89607	4.931794	24.07327	3.52E‐09	Gleason score	11.01887943	4.900614	24.77561	6.45E‐09
ECMG risk score	1.041191	1.004977	1.078709	0.025427	ECMG risk score	1.036041726	1.003167	1.069994	0.031387

**Table 5:** Results of univariate and multivariate Cox regression analyses for conventional clinicopathological parameter and ECMG signature risk score in DKFZ cohort. ECMG, epithelial cell marker gene; PSA, prostate‐specific antigen; T stage, tumour stage.

Univariate Cox regression					Multivariate Cox regression				
Id	HR	HR.95 L	HR.95H	*P* value	Id	HR	HR.95 L	HR.95H	*P* value
Pathological T stage	4.917352	2.028672	11.9193	0.000422	Pathological T stage	0.760864	0.228159	2.537326	0.656502
PSA	12.33637	3.598123	42.29598	6.42E‐05	PSA	6.228881	1.502468	25.82348	0.0117
Gleason score	10.76351	4.489437	25.80572	1.00E‐07	Gleason score	3.431298	1.12451	10.47016	0.030301
ECMG risk score	1.141974	1.084378	1.202629	4.96E‐07	ECMG risk score	1.100629	1.037253	1.167878	0.001531

**Table 6:** Results of univariate and multivariate Cox regression analyses for conventional clinicopathological parameter and ECMG signature risk score in GSE70768 cohort. Age, age at diagnosis; ECMG, epithelial cell marker gene; N stage, lymph node status (N0 = without lymph node metastasis; N1 = with lymph node metastasis); PSA, prostate‐specific antigen; T stage, tumour stage.

Univariate Cox regression					Multivariate Cox regression				
Id	HR	HR.95 L	HR.95H	*P* value	Id	HR	HR.95 L	HR.95H	*P* value
Age	0.736515	0.247126	2.195049	0.583079	Age	0.815202	0.234229	2.837194	0.748132
Pathological T stage	2.077162	0.460026	9.379053	0.341898	Pathological T stage	1.76818	0.365815	8.546567	0.478325
Pathological N stage	2.254584	0.496405	10.23994	0.292387	Pathological N stage	1.97755	0.363306	10.76423	0.430263
PSA	0.738375	0.20308	2.68464	0.645142	PSA	0.419218	0.102784	1.709831	0.225474
Gleason score	8.852992	2.347368	33.38866	0.001283	Gleason score	6.423792	1.539267	26.80828	0.010721
ECMG risk score	1.051409	0.999344	1.106186	0.053034	ECMG risk score	1.047737	0.990988	1.107736	0.100728

**Table 7:** Results of univariate and multivariate Cox regression analyses for conventional clinicopathological parameter and ECMG signature risk score in E‐MTAB‐6128 cohort. Age, age at diagnosis; ECMG, epithelial cell marker gene; PSA, prostate‐specific antigen.

Univariate Cox regression					Multivariate Cox regression				
Id	HR	HR.95 L	HR.95H	*P* value	Id	HR	HR.95 L	HR.95H	*P* value
Age	1.238353	0.267426	5.734358	0.784562	Age	1.108892	0.216627	5.676308	0.901267
PSA	2.859212	0.814541	10.03644	0.101051	PSA	4.043797	0.987701	16.55591	0.052046
Gleason score	18.62878	4.270013	81.27173	9.97E‐05	Gleason score	18.09657	3.403796	96.21195	0.000682
ECMG risk score	1.054576	0.992579	1.120446	0.085612	ECMG risk score	1.025841	0.952316	1.105042	0.501363

### 3.7 The immune landscape and drug response of the ECMG‐based signature

Although individual stromal and immune cell scores did not differ significantly between the high‐ and low‐risk groups of PCa patients in the TCGA‐PRAD cohort, the overall stromal and immune cell scores (i.e., the ESTIMATE score) were significantly different, with higher scores observed in the high‐risk group. This suggests that the higher overall scores could reflect a more aggressive tumour environment, underscoring their potential as indicators of increased risk in these patients (Fig. S8).

**Fig. 7:**
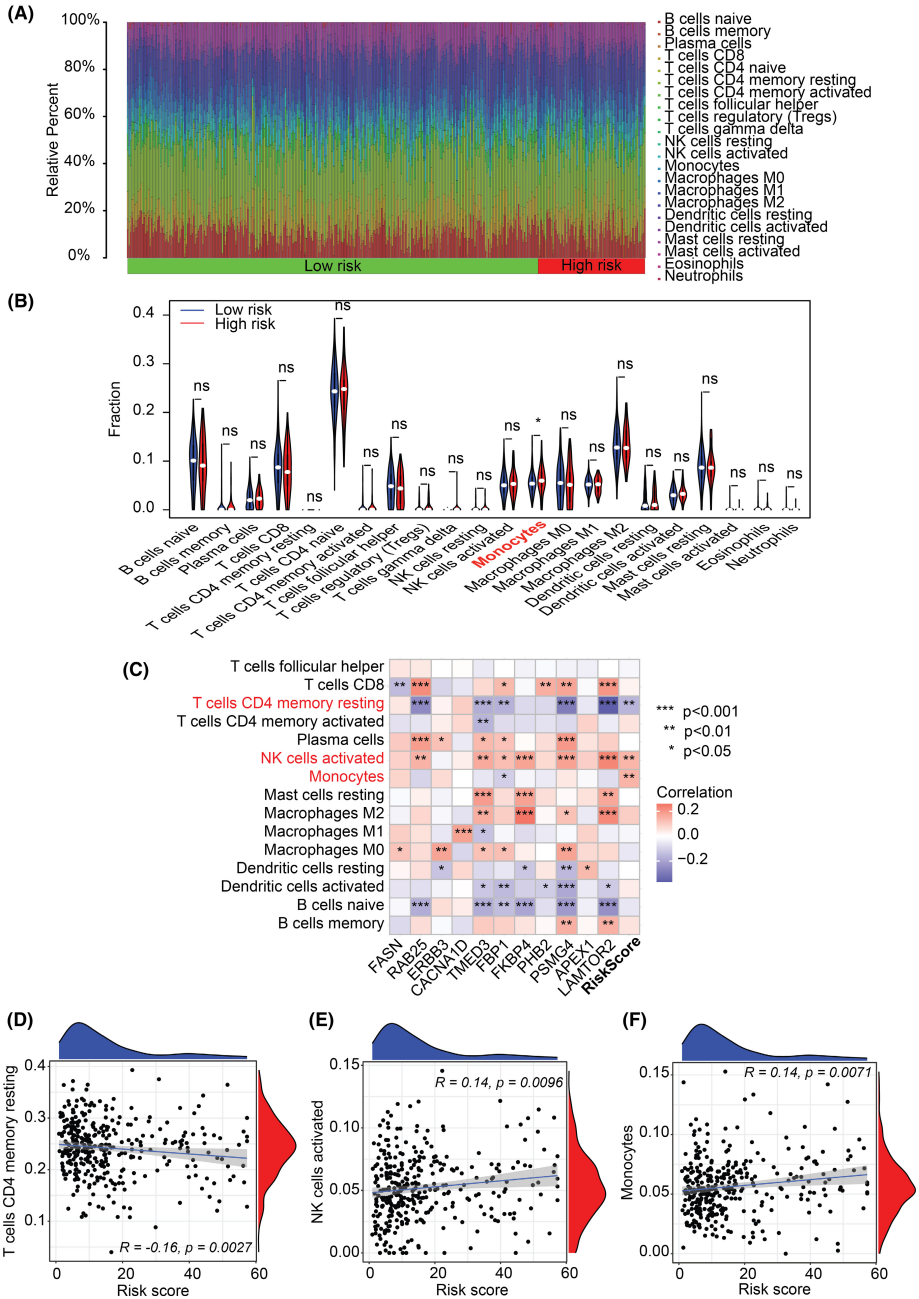
Immune cell infiltration and differences between risk groups in the TCGA‐PRAD Cohort. (A) Distribution of all immune cells in each sample. (B) Violin plot illustrating the fraction of each immune cell type in high‐risk and low‐risk patient groups. Statistical comparisons were performed using an unpaired Wilcoxon test. Immune cell types highlighted in red denote significant differences between the groups. (C) Heatmap showing Spearman correlations between immune cell types and the epithelial cell marker gene (ECMG) signature, including its constituent genes. Immune cell types highlighted in red denote significant correlations with the signature risk scores. Spearman correlation analyses between the ECMG signature risk score and three immune cell types: CD4 memory resting T cells (D), activated NK cells (E) and monocytes (F). Error bars represent the 95% confidence intervals. Significance levels are indicated as follows: *P < 0.05, **P < 0.01, ***P < 0.001, ns, not significant. CD, cluster of differentiation; NK, natural killer; PRAD, prostate adenocarcinoma; TCGA, The Cancer Genome Atlas.

The CIBERSORT analysis revealed that T cells and macrophages are the most prevalent cell types in the TCGA‐PRAD samples (Fig. 7A). Additionally, a significantly higher proportion of monocytes was found in patients of the high‐risk group compared to those in the low‐risk group (Fig. 7B). The ECMG‐based signature risk score exhibited a significantly positive association with activated NK cells and monocytes, whereas a significant negative association was observed between the signature risk score and CD4 memory resting T cells (Fig. 7C–F).

The database Genomics of Drug Sensitivity in Cancer was used to identify potentially effective chemotherapeutic agents for patients stratified within the high‐risk group (i.e., worse BCRFS). The predictive analysis of drug response identified 10 agents and demonstrated that, compared to low‐risk patients, high‐risk patients were significantly more sensitive to the drug WIKI4 (Fig. S9). This suggests that WIKI4 may be effective in treating individuals with a poorer prognosis, indicating its potential as a targeted therapeutic option for this subgroup.

### 3.8 Identification of potential non‐invasive biomarkers in blood‐based transcriptomic data

**Fig. 8:**
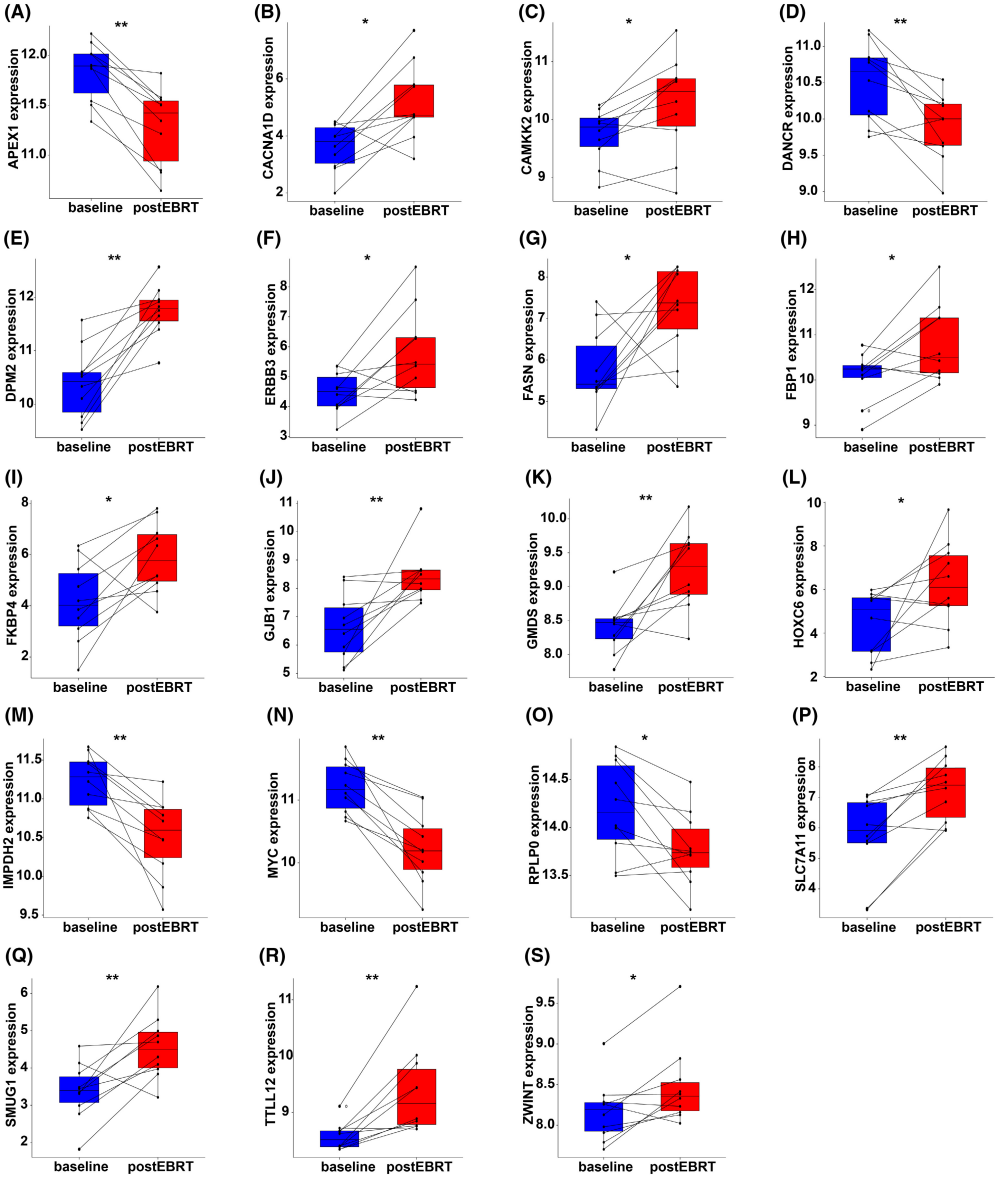
Differential expression analysis of prostate cancer associated genes in blood samples post‐external beam radiation therapy (EBRT) in GSE30174. (A–S) Boxplots comparing the expression levels of genes associated with prostate cancer, between blood samples collected 4 weeks after localised EBRT (postEBRT) and matched normal blood samples prior to EBRT (baseline). Statistical comparisons were performed using a paired Wilcoxon test. For each boxplot, lines connect each baseline sample to its matched postEBRT sample. Significance levels are indicated as follows: *P < 0.05, **P < 0.01.

Whole blood samples (GSE30174) derived from localised PCa patients before and 4 weeks after EBRT treatment revealed significant changes in ECMG‐based signature genes. *APEX1* was significantly downregulated post‐EBRT (*P* < 0.01; Fig. 8A). Conversely, *CACNA1D*, *ERBB3*, *FASN*, *FBP1 and FKBP4* showed significant increases post‐EBRT (*P* < 0.05; Fig. 8B,F–I, respectively).

**Fig. 9:**
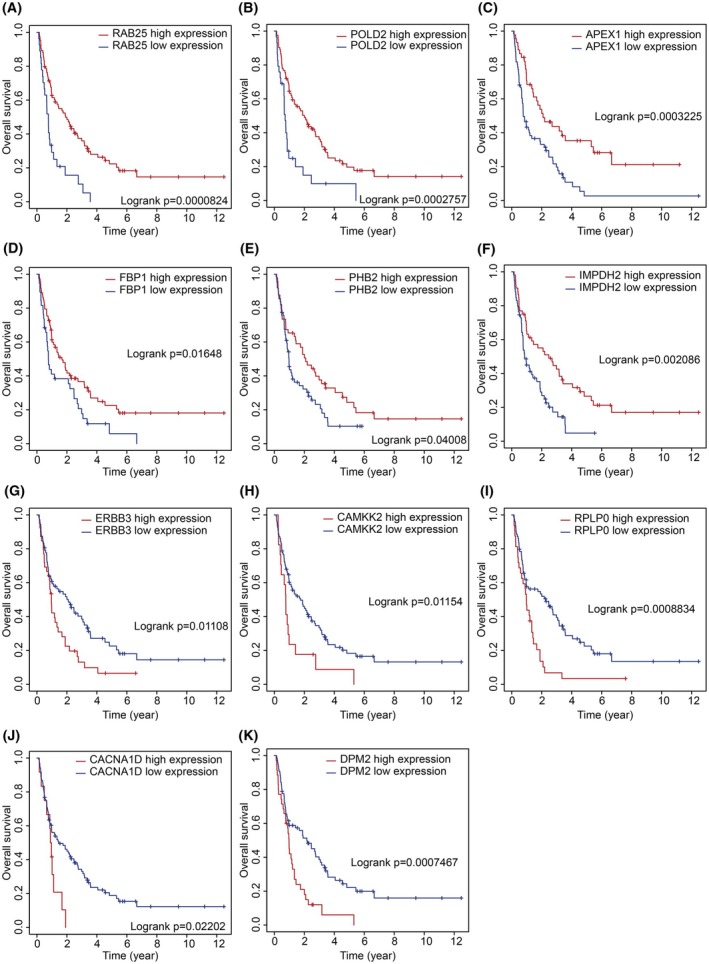
Kaplan–Meier survival analysis of candidate epithelial marker genes in prostate cancer in GSE53922. (A–K) Kaplan–Meier survival curves complemented by log‐rank tests for each of the overlapping genes associated with prostate cancer, demonstrating their potential prognostic significance.

In the PBMC cohort (GSE53922) of CRPC patients, KM analysis revealed that the genes *RAB25* (*P* < 0.0001), *FBP1*, *PHB2*, *ERBB3*, *CACNA1D* (each *P* < 0.05) and *APEX1* (*P* < 0.001) could significantly stratify patients into high‐ and low‐risk groups based on their overall survival (Table S6). Specifically, patients with high expression levels of *RAB25*, *APEX1*, *FBP1* and *PHB2* had improved overall survival (Fig. 9A,C,D,E, respectively), while elevated expressions of *ERBB3* and *CACNA1D* were associated with poorer overall survival (Fig. 9G,J, respectively).

## 4 Discussion

The clinical, molecular and morphological heterogeneity of prostate cancer (PCa) complicates the effective diagnosis, prognosis and treatment strategies [46]. Epithelial cells, which form the basis of most human organs and are involved in about 90% of all human cancers, play an important role in maintaining tissue architecture through properties like intercellular adhesion, apical‐basal polarity and regulated proliferation and disruption of these is a hallmark of cancer progression [47]. Epithelial cells are involved in both the initiation and progression of PCa. Recent studies have highlighted the significant role of basal epithelial cells in prostate development, oncogenesis and the emergence of therapeutic resistance, making them potential targets for innovative treatments [48]. BCRFS is a critical endpoint in PCa management, serving as an early indicator of the disease progression following treatments like RP or radiotherapy. Timely detection of BCR is essential for effective intervention and significantly influences long‐term outcomes [49]. Recent advancements in sequencing technologies and bioinformatics tools have enabled the integration of bulk RNA‐seq and scRNA‐seq data, providing a deeper understanding of the molecular characteristics and heterogeneity within prostate tumours, enhancing prognostic power for patients' BCRFS and impacting immunotherapy and other treatment responses [50, 51, 52]. Despite improvements in prognosis based on cell‐specific markers, including macrophages [23] and cancer‐associated fibroblasts [53, 54], the investigation of epithelial markers as prognostic indicators for BCRFS remains underexplored. Addressing this gap is essential for advancing the development of effective therapies and improving risk stratification models in PCa. This study aimed to bridge this gap by developing a robust PCa‐associated ECMG signature through an integrative approach of scRNA‐seq and bulk RNA‐seq, enhanced by machine learning bioinformatics, to refine the accuracy of BCRFS prognosis in PCa patients and therefore better manage the disease.

Bulk RNA‐seq and scRNA‐seq offer complementary insights into the transcriptomic landscape of PCa. Bulk RNA‐seq provides a broad overview, ideal for identifying dominant expression trends and potential biomarkers across large sample sets [50]. In contrast, scRNA‐seq offers detailed views of cellular heterogeneity, crucial for uncovering distinct cellular identities and interactions within the tumour microenvironment [55]. The integration of these methods allows for a comprehensive understanding of gene expression changes and supports the development of targeted therapies by validating bulk RNA‐seq findings at the cellular level [56].

Recent studies have demonstrated the importance of epithelial cell markers in understanding PCa's molecular landscape. For example, the epithelial cell adhesion molecule (EpCAM) is significantly upregulated in PCa tissues, correlating with tumour occurrence, progression and particularly bone metastasis [57]. Shifts in adhesion molecules like the transition from E‐cadherin to N‐cadherin signify epithelial to mesenchymal transition, associated with more aggressive tumours and poorer prognosis [58].

A comprehensive meta‐analysis of four large‐scale PCa cohorts identified 33 out of 48 pre‐defined genes consistently overexpressed in PCa tumours, which aligns with our previous findings [30]. By cross‐referencing these genes with marker genes of various cell types, we observed that the majority were significant epithelial markers, suggesting the importance of epithelial cells in PCa. Pathway analysis revealed significant enrichment of these markers in the ‘AMPK signaling pathway’. Previous research has reported that AMPK activation can inhibit androgen receptor (AR) transcriptional activity, which is crucial for PCa progression, thus indicating AMPK as a tumour suppressor in PCa [59]. Furthermore, inhibiting AR function enhances AMPK activation, subsequently reducing PCa cell growth [60].

Although a single gene may not suffice for predictive accuracy on its own, its integration with other genes in a multi‐gene model can significantly enhance predictive capability [61]. Previous studies have developed a variety of gene‐based signatures for cancer prognosis and have conducted systematic and unbiased evaluations of these signatures across various cancer types such as breast cancer [62, 63], lung cancer [64] and ovarian cancer [65]. Over recent decades, many gene signatures have been widely applied in cancer genomics, particularly in the development of prognostic models for high‐dimensional transcriptomics data. A previous study compared a total of 30 published prostate cancer (PCa) gene signatures and highlighted their potential to improve prognostic accuracy for BCRFS using various machine learning models [29]. However, systematic approaches for using epithelial markers as prognostic signatures remain underexplored. In this study, we used 97 machine learning approaches and identified the RSF model as the most effective for predicting BCRFS in PCa patients across five large‐scale public PCa cohorts. This epithelial cell marker gene (ECMG) based prognostic signature overall outperformed 68 published PCa signatures and even some commercial tests, including Decipher, Oncotype DX GPS and Prolaris [66]. The ECMG signature also demonstrated robust predictive capabilities for 1‐, 3‐ and 5‐year BCRFS and was effective in stratifying patients into high‐ and low‐risk groups, with high‐risk patients showing poorer outcomes. Further validation of the ECMG signature for prognosis after radical radiotherapy in the Belfast cohort enhanced its clinical utility, showing poorer outcomes for high‐risk patients in terms of both BCRFS and distant metastasis‐free survival. The ECMG signature indicated an improvement in the accuracy of individual BCR risk assessments compared to traditional clinicopathological factors. It may be considered an independent prognostic factor, particularly in large PCa cohorts. These results suggest the potential clinical utility of the ECMG‐based signature model in improving the prognosis of PCa patients.

Among the genes in our ECMG‐based signature model, *TMED3* (transmembrane emp24 protein transport domain containing 3) was consistently found to be overexpressed, both in our work and in the literature. It has also been identified as an epithelial marker and suggested as a potential therapeutic target for PCa [24]. Another study confirmed its overexpression in PCa tumours compared to normal counterparts, regulated by the *ERG* oncogene and androgens and proposed it as a promising drug target in PCa [67]. *FASN* (fatty acid synthase) has been reported to exhibit elevated expression in both androgen‐dependent and ‐independent PCa and overexpression of *FASN* is associated with resistance to chemotherapy [68]. *FASN* overexpression also correlates with poor BCRFS in PCa, suggesting its potential as a prognostic marker for the disease [69]. Inhibition of *FASN* has demonstrated the ability to arrest PCa growth both *in vitro* and *in vivo* [70, 71, 72] and shown promise in targeted cancer therapy [73, 74, 75]. Furthermore, inhibiting *FASN* enhances the outcomes of radiotherapy by redistributing the cell cycle and downregulating AR signalling in LNCaP cells [76].

In metastatic castration‐resistant prostate cancer (mCRPC), *FASN* is highly expressed and found in 87% of metastases. Among patients treated with enzalutamide or abiraterone, *FASN*/AR‐V7 double‐positive metastases were present in 77% of cases. A promising *FASN* inhibitor, IPI‐9119, has shown potential in preclinical studies by significantly reducing cell growth, causing cell cycle arrest and triggering apoptosis in prostate cancer cells [77]. These findings suggest that IPI‐9119 could be valuable in future clinical trials, particularly as an adjuvant or alternative therapeutic approach for patients resistant to current AR‐targeted therapies. *APEX1* is another key target, as it is overexpressed in ERG‐negative prostate cancers [78] and is significantly higher in prostate cancer tissues compared to noncancerous controls. Redox‐specific small‐molecule inhibitors of *APEX1*, such as APX3330 and APX2009, have shown the ability to suppress prostate cancer cell growth and induce cell cycle arrest in preclinical studies [79].

For a gene signature to have real clinical utility, it would ideally be accessible from minimally‐invasive tissue samples. Our investigation of blood‐based datasets GSE30174 and GSE53922 from PCa patients revealed that 4 genes from our ECMG signature, including *FBP1*, *ERBB3*, *CACNA1D* and *APEX1*, were not only significantly altered in localised PCa patients post‐EBRT but also held prognostic value in stratifying CRPC patients into high‐ and low‐risk categories for overall survival. These results highlight their potential utility as non‐invasive biomarkers for tracking treatment response, prognostication and guiding treatment strategies in PCa patients. Although direct evidence supporting these genes as non‐invasive biomarkers for PCa is lacking, and they have yet to be clinically utilised, our findings could inform future research and potential clinical trials. Lastly, despite robust performance of our signature in predicting BCRFS in PCa patients, all cohorts analysed were retrospective. Therefore, prospective studies that include diverse ethnic groups and multi‐centre collaborations are crucial for further validation of our signature.

## 5 Conclusions

This study identified a set of ECMGs that showed consistent upregulation in prostate tumours and prognostic significance for BCRFS in patients diagnosed with PCa. Utilising advanced machine learning algorithms, we developed an 11‐ECMG‐based signature that not only improves the predictive accuracy but also enhances risk stratification for patients' BCRFS. This signature demonstrated an overall superior predictive power for BCRFS compared to some published signatures and proved to be an independent prognostic predictor over conventional parameters in larger cohorts. Furthermore, our investigation into non‐invasive biomarkers from blood samples indicates that some ECMG signature genes may hold potential for monitoring treatment responses for localised patients and assisting in the stratification of overall survival risks among castration‐resistant patients.

## Conflict of interest

LWH is founder, director and chief scientific officer of SENISCA Ltd. SENISCA's commercial activities have no bearing on the content of this publication.

## Author contributions

ZM carried out the analysis and wrote the draft. LWH provided overall oversight, managed the project and finalised the draft.

## Peer review

The peer review history for this article is available at https://www.webofscience.com/api/gateway/wos/peer‐review/10.1002/1878‐0261.13804.

## Supporting information


**Fig. S1.** Meta‐analysis of 33 differentially expressed genes in prostate cancer.
**Fig. S2.** Overlap analysis.
**Fig. S3.** Expression patterns of prostate cancer associated epithelial cell marker genes.
**Fig. S4.** Construction and predictive assessment of the epithelial cell marker gene signature using machine learning algorithms.
**Fig. S5.** Waterfall map for displaying landscape of somatic mutations.
**Fig. S6.** Comparative analysis of epithelial cell marker gene‐based signature against clinicopathological parameters.
**Fig. S7.** Box plot between epithelial cell marker gene (ECMG) signature risk score and different clinicopathological parameters across five cohorts.
**Fig. S8.** ESTIMATE analysis of stromal, immune and overall cell scores in different patient risk groups in TCGA‐PRAD.
**Fig. S9.** Drug response prediction analysis.
**Table S1.** Results of meta‐analysis.
**Table S2.** Intersection of epithelial cell marker genes and differentially expressed genes in prostate tumour from meta‐analysis.
**Table S3.** Results of Kaplan–Meier survival analysis with log‐rank test for the 17 epithelial cell marker genes in prostate cancer.
**Table S4.** Construction and validation of epithelial cell marker gene based signatures using machine learning algorithms across TCGA‐PRAD, Taylor (GSE21034), Cambridge (GSE70768), CIT (E‐MTAB‐6128) and DKFZ cohorts.
**Table S5.** Overview of 11 signature genes and their associations with prostate cancer.
**Table S6.** Results of Kaplan–Meier survival analysis with log‐rank test on the panel genes in GSE53922.

## Data Availability

Datasets GSE193337, GSE30174 and GSE53922 can be identified and downloaded from the GEO database (https://www.ncbi.nlm.nih.gov/geo/) under the corresponding accession number. Pre‐processed datasets for TCGA‐PRAD, GSE21034, GSE70768, E‐MTAB‐6128 and DKFZ were retrieved from the PCaDB database (http://bioinfo.jialab‐ucr.org/PCaDB/). Essential code and data that support the findings of this study can be required upon reasonable request from the corresponding author.
