# Supplementary material for: Integration of single‐cell and bulk RNA‐sequencing data reveals the prognostic potential of epithelial gene markers for prostate cancer
Source: Mol Oncol. 2025 Feb 19;19(6):1811–35. doi: 10.1002/1878-0261.13804 (PMC12161480; doi:10.1002/1878-0261.13804)
Supplement: Supplementary file 1 — Fig. S1. Meta‐analysis of 33 differentially expressed genes in prostate cancer. Fig. S2. Overlap analysis. Fig. S3. Expression patterns of prostate cancer associated epithelial cell marker genes. Fig. S4. Construction and predictive assessment of the epithelial cell marker gene signature using machine learning algorithms. Fig. S5. Waterfall map for displaying landscape of somatic mutations. Fig. S6. Comparative analysis of epithelial cell marker gene‐based signature against clinicopathological parameters. Fig. S7. Box plot between epithelial cell marker gene (ECMG) signature risk score and different clinicopathological parameters across five cohorts. Fig. S8. ESTIMATE analysis of stromal, immune and overall cell scores in different patient risk groups in TCGA‐PRAD. Fig. S9. Drug response prediction analysis. Table S1. Results of meta‐analysis. Table S2. Intersection of epithelial cell marker genes and differentially expressed genes in prostate tumour from meta‐analysis. Table S3. Results of Kaplan–Meier survival analysis with log‐rank test for the 17 epithelial cell marker genes in prostate cancer. Table S4. Construction and validation of epithelial cell marker gene based signatures using machine learning algorithms across TCGA‐PRAD, Taylor (GSE21034), Cambridge (GSE70768), CIT (E‐MTAB‐6128) and DKFZ cohorts. Table S5. Overview of 11 signature genes and their associations with prostate cancer. Table S6. Results of Kaplan–Meier survival analysis with log‐rank test on the panel genes in GSE53922. [file MOL2-19-1811-s001.docx]

**Supplementary Figures**

**
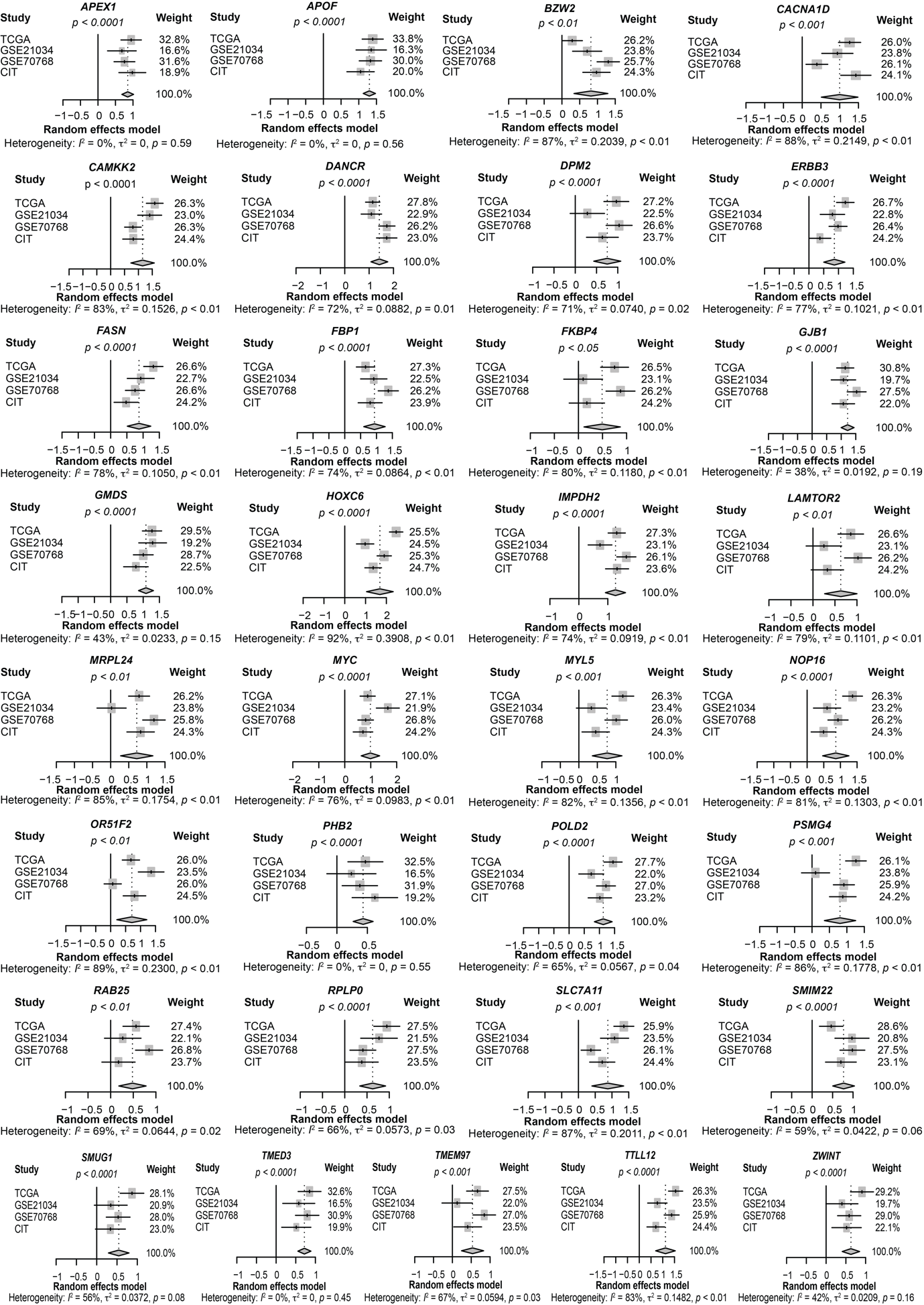
**

*Figure legend in the next page.*

**Supplementary Figure S1: Meta-analysis of 33 differentially expressed genes in prostate cancer.**

Forest plot showing the effect sizes and confidence intervals for each of the 33 genes identified as differentially expressed through meta-analysis.

**
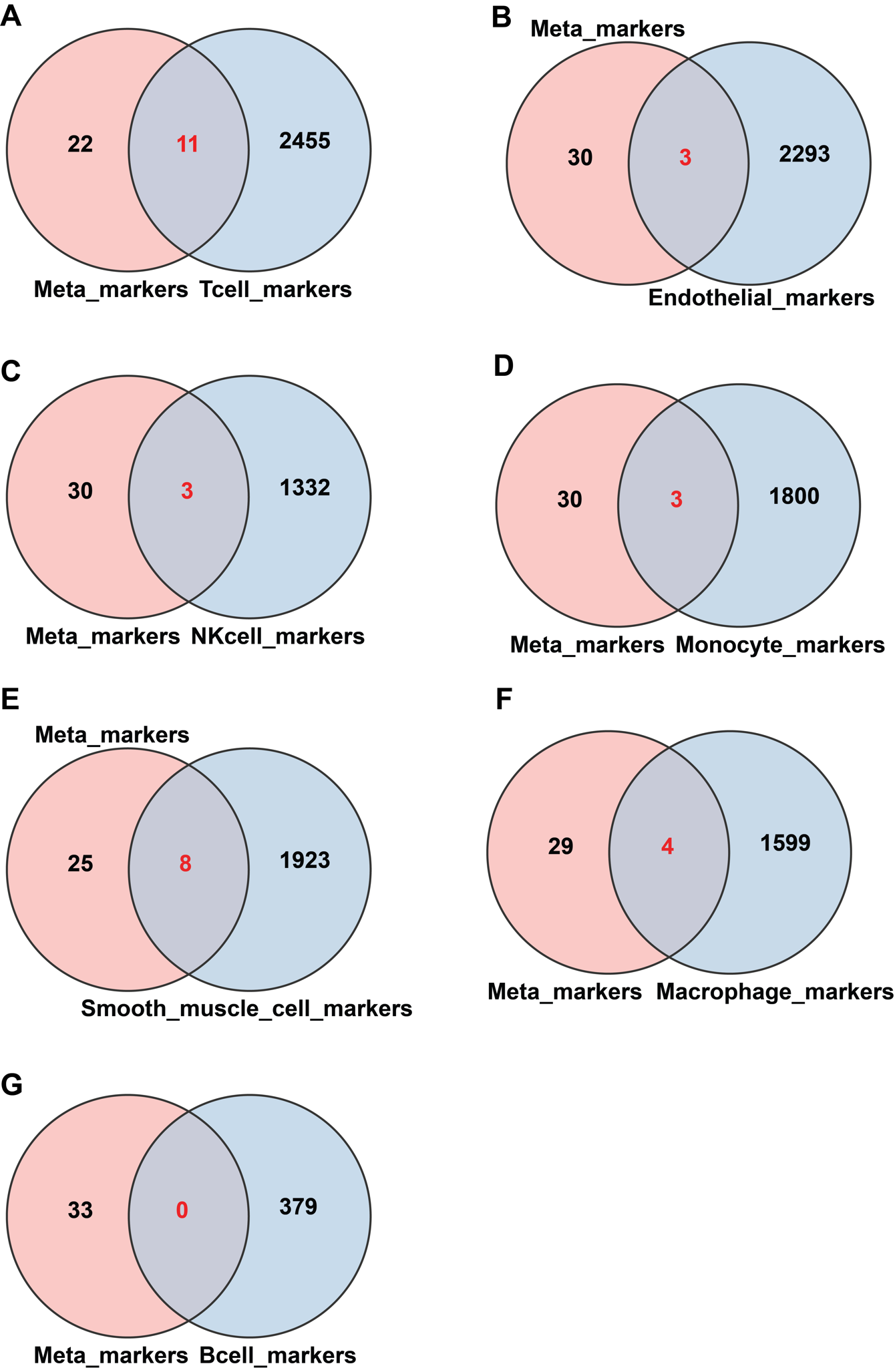
**

*Figure legend in the next page.*

**Supplementary Figure S2: Overlap Analysis.**

Venn diagrams showing the number of overlapping genes between upregulated genes in prostate cancer, identified by meta-analysis, and cell type specific marker genes in: **(A)** T cells, **(B)** endothelial cells, **(C)** natural killer (NK) cells, **(D)** monocytes, **(E)** smooth muscle cells, **(F)** macrophages, and **(G)** B cells.


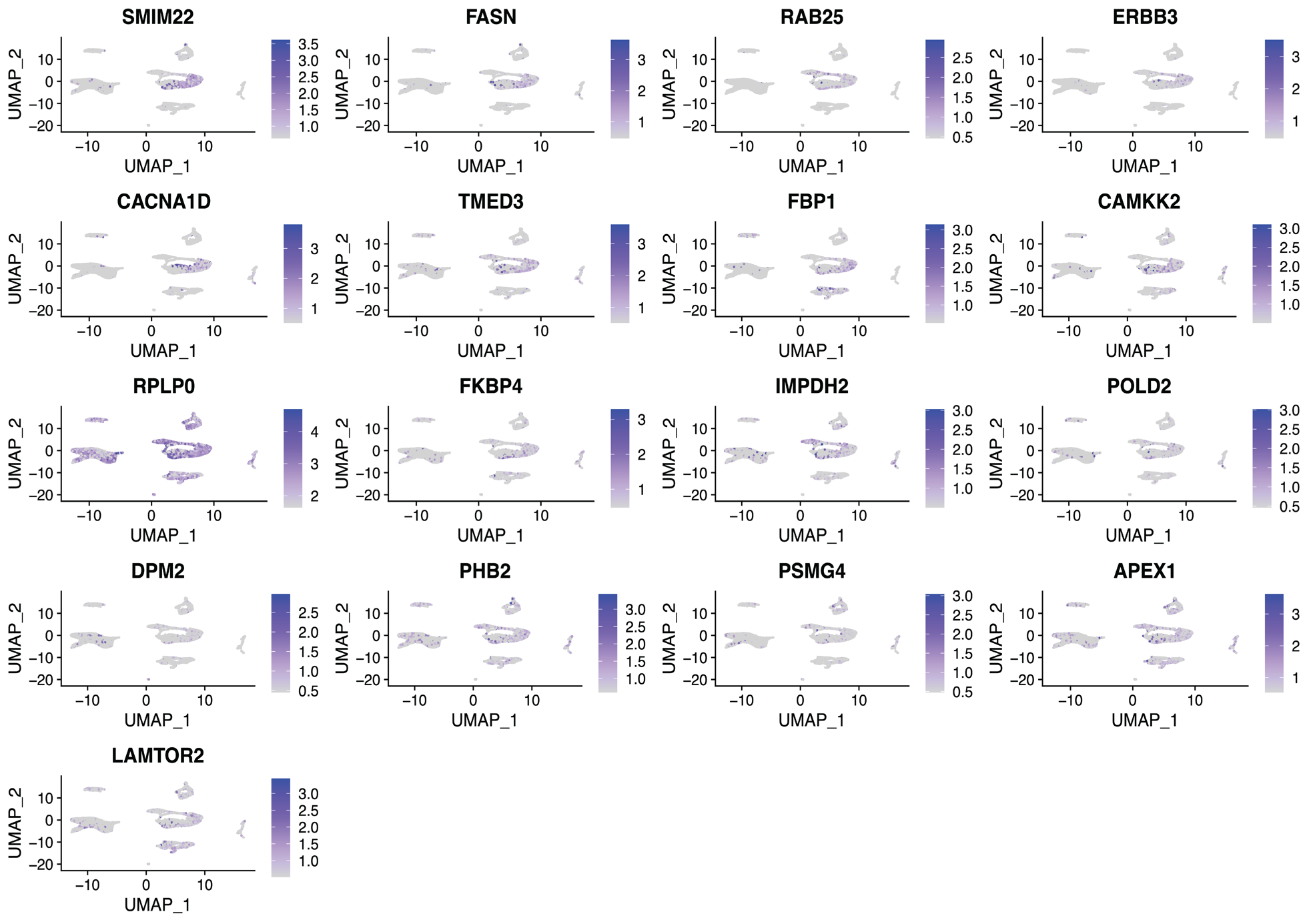


*Figure legend in the next page.*

**Supplementary Figure S3: Expression patterns of prostate cancer associated epithelial cell marker genes.**

Uniform manifold approximation and projection (UMAP) plots showing the expression patterns of 17 epithelial cell marker genes intersecting with prostate cancer-associated genes identified through meta-analysis.


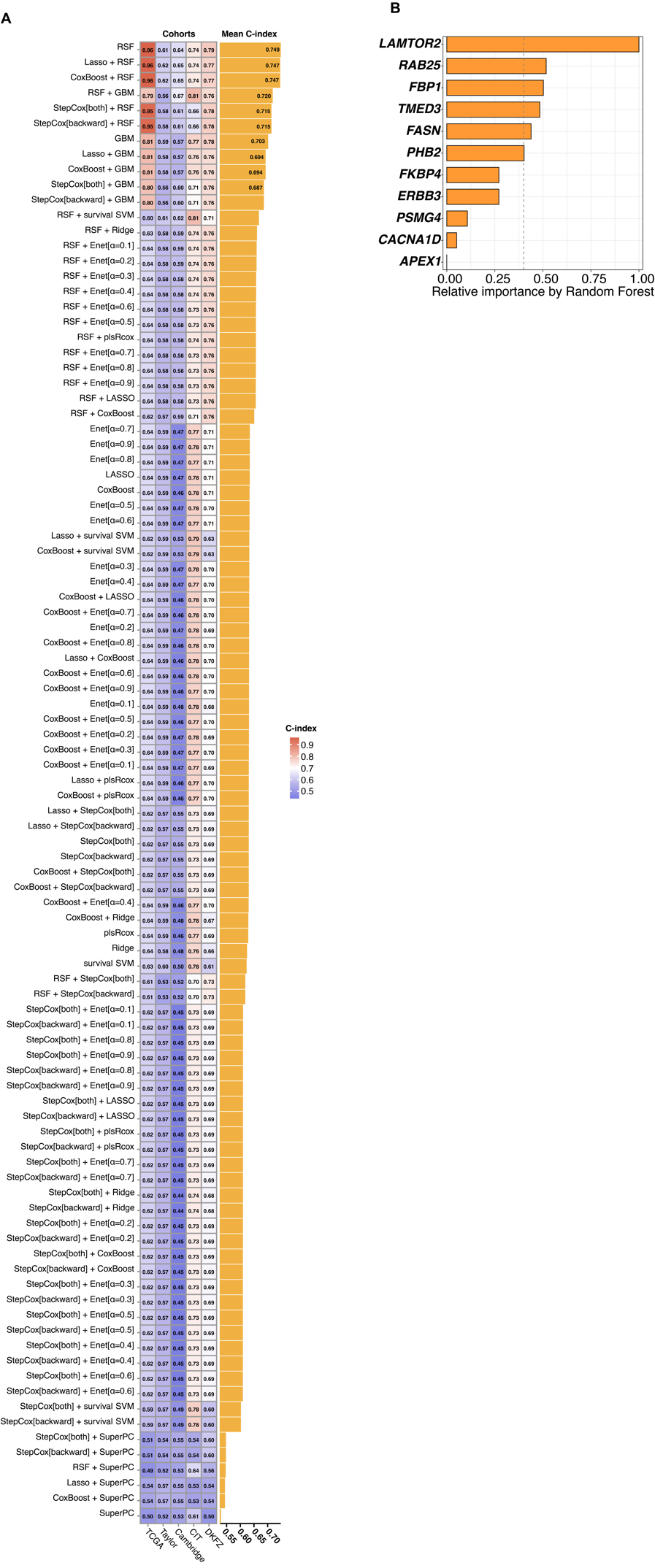


*Figure legend in the next page.*

**Supplementary Figure S4: Construction and predictive assessment of the epithelial cell marker gene signature using machine learning algorithms.**

**(A)** Performance of the gene signature in predicting biochemical recurrence-free survival (BCRFS) in prostate cancer patients across TCGA-PRAD, Taylor (GSE21034), Cambridge (GSE70768), CIT (E-MTAB-6128), and DKFZ cohorts, evaluated using 97 different machine learning models. The performance is measured by the concordance index (C-index) for each model in each cohort. The top 10 models are labelled with their corresponding C-index values. **(B)** Relative importance of the genes in the random survival forest model, with an importance threshold set at 0.4. RSF: random survival forest; LASSO: least absolute shrinkage and selection operator; GBM: generalised boosted regression modelling; Enet: elastic net; plsRcox: partial least squares regression for Cox; SVM: support vector machine; SuperPC: supervised principal components.


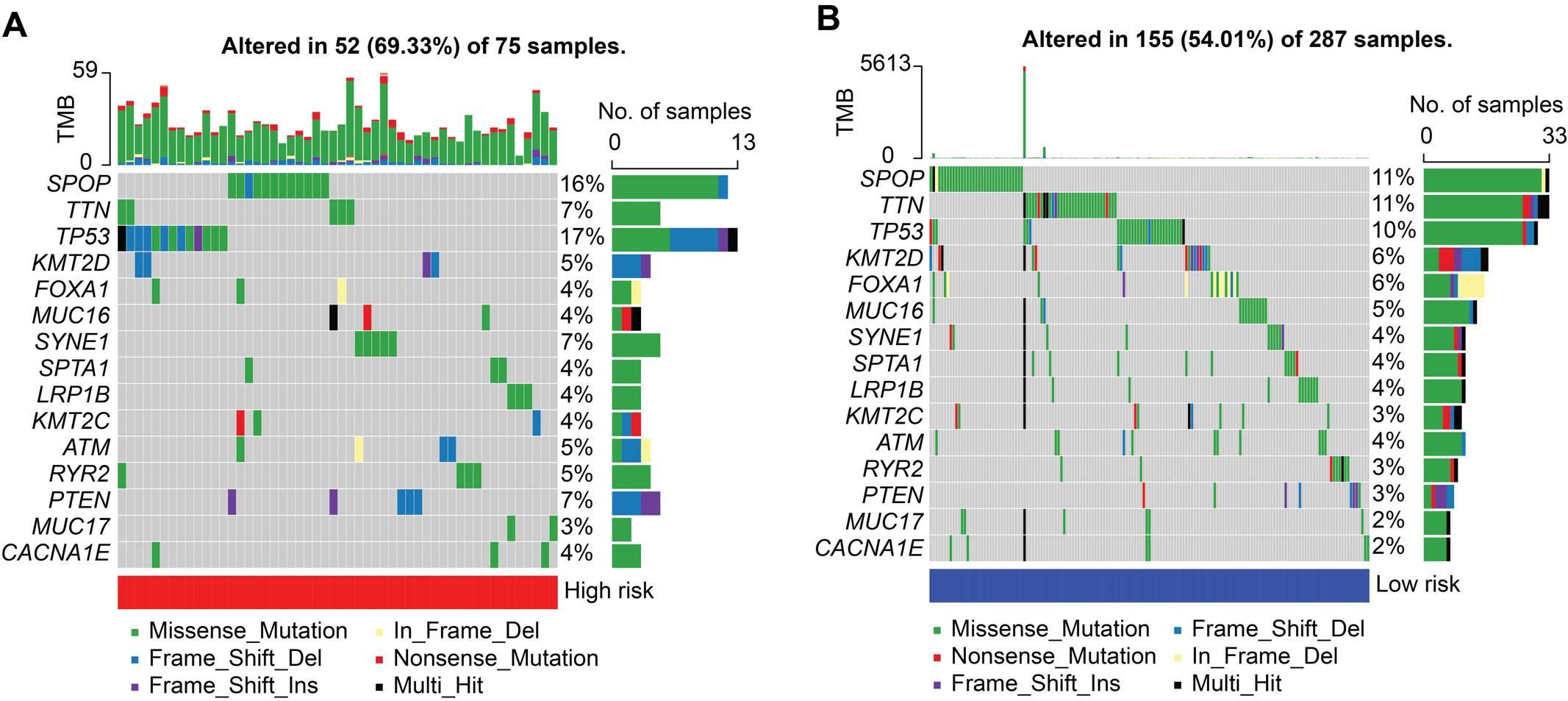


**Supplementary Figure S5: Waterfall map for displaying landscape of somatic mutations.**

Top 15 mutated gene are shown in the high-risk group **(A)** and low-risk group **(B)** stratified by the epithelial cell marker gene based signature. TMB: tumour mutation burden; Del: deletion; Ins: insertion.


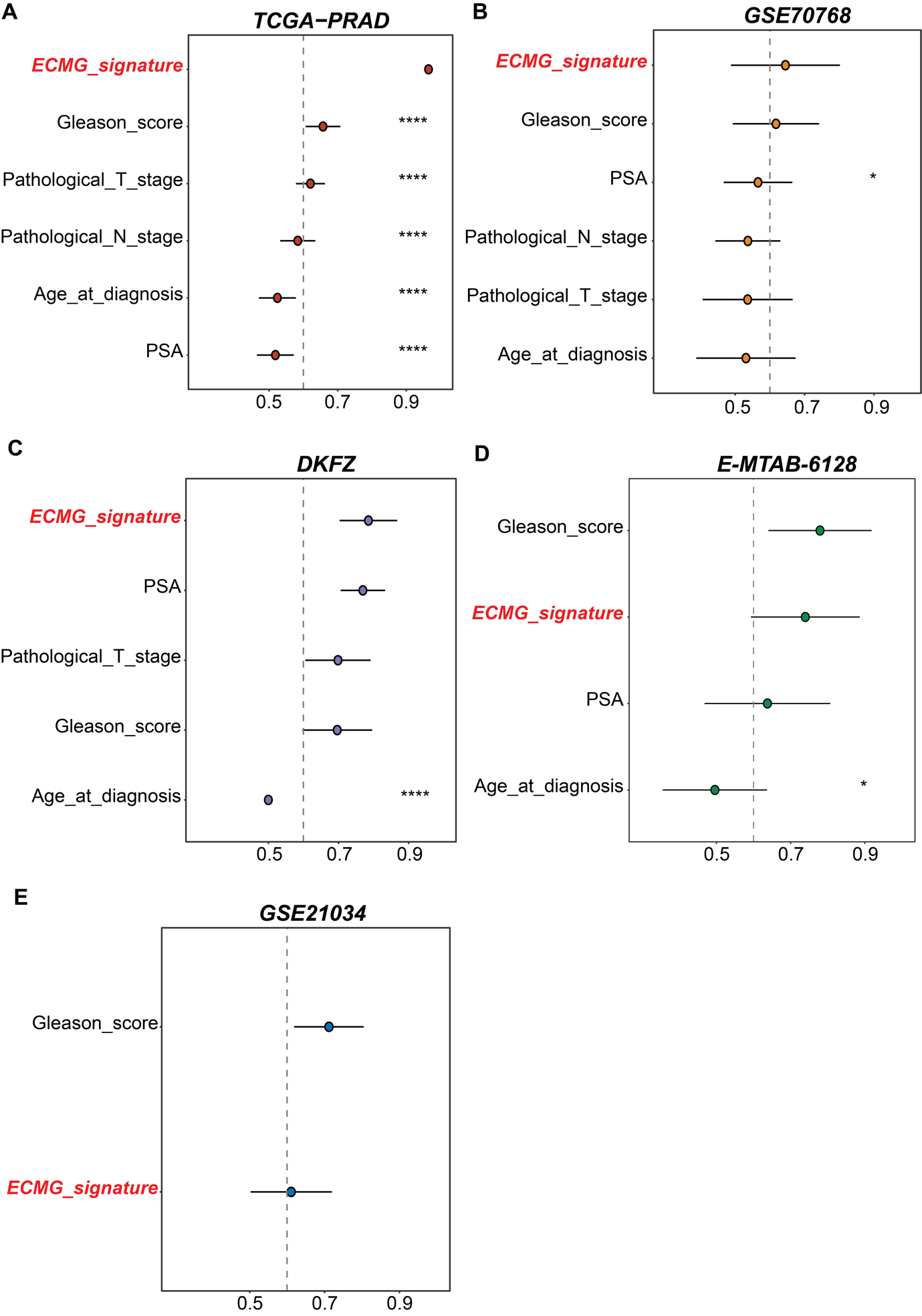


*Figure legend in the next page.*

**Supplementary Figure S6: Comparative analysis of epithelial cell marker gene-based signature against clinicopathological parameters.**

Concordance index (C-index) values for each variable were calculated and compared to assess their predictive power in **(A)** TCGA-PRAD, **(B)** GSE70768, **(C)** DKFZ, **(D)** E-MTAB-6128, and **(E)** GSE21034 cohorts. TCGA: The Cancer Genome Atlas; PRAD: prostate adenocarcinoma; ECMG: epithelial cell marker gene.


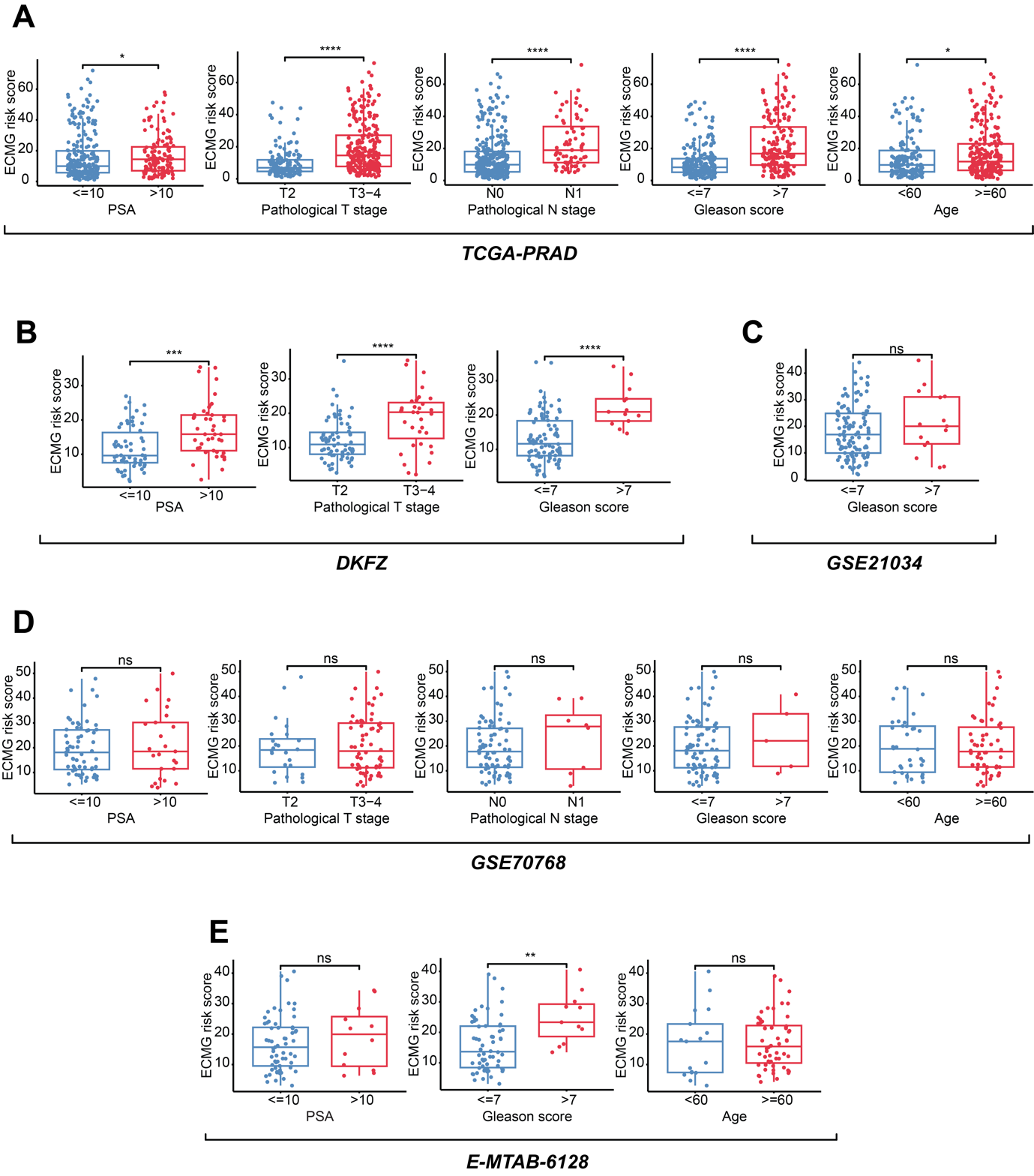


*Figure legend in the next page.*

**Supplementary Figure S7: Box plot between epithelial cell marker gene (ECMG) signature risk score and different clinicopathological parameters across five cohorts.**

**(A)** Boxplots showing ECMG signature risk score in different prostate-specific antigen (PSA) levels (<=10 vs. >10), pathological T stages (T2 vs. T3-4), pathological N stages (N0 vs. N1), Gleason scores (<=7 vs. >7), and age groups (<60 vs. >=60) in TCGA-PRAD cohort. **(B)** Boxplots showing ECMG signature risk score in different PSA levels (<=10 vs. >10), pathological T stages (T2 vs. T3-4), and Gleason scores (<=7 vs. >7) in DKFZ. **(C)** Boxplots showing ECMG signature risk score in different Gleason scores (<=7 vs. >7) in GSE21034 cohort. **(D)** Boxplots showing ECMG signature risk score in different PSA levels (<=10 vs. >10), pathological T stages (T2 vs. T3-4), pathological N stages (N0 vs. N1), Gleason scores (<=7 vs. >7), and age groups (<60 vs. >=60) in GSE70768 cohort. **(E)** Boxplots showing ECMG signature risk score in different PSA levels (<=10 vs. >10), Gleason scores (<=7 vs. >7), and age groups (<60 vs. >=60) in E-MTAB-6128 cohort. TCGA: The Cancer Genome Atlas; PRAD: prostate adenocarcinoma; Significance levels are indicated as follows: *p < 0.05, **p < 0.01, ***p < 0.001, ****p < 0.0001, ns = not significant.


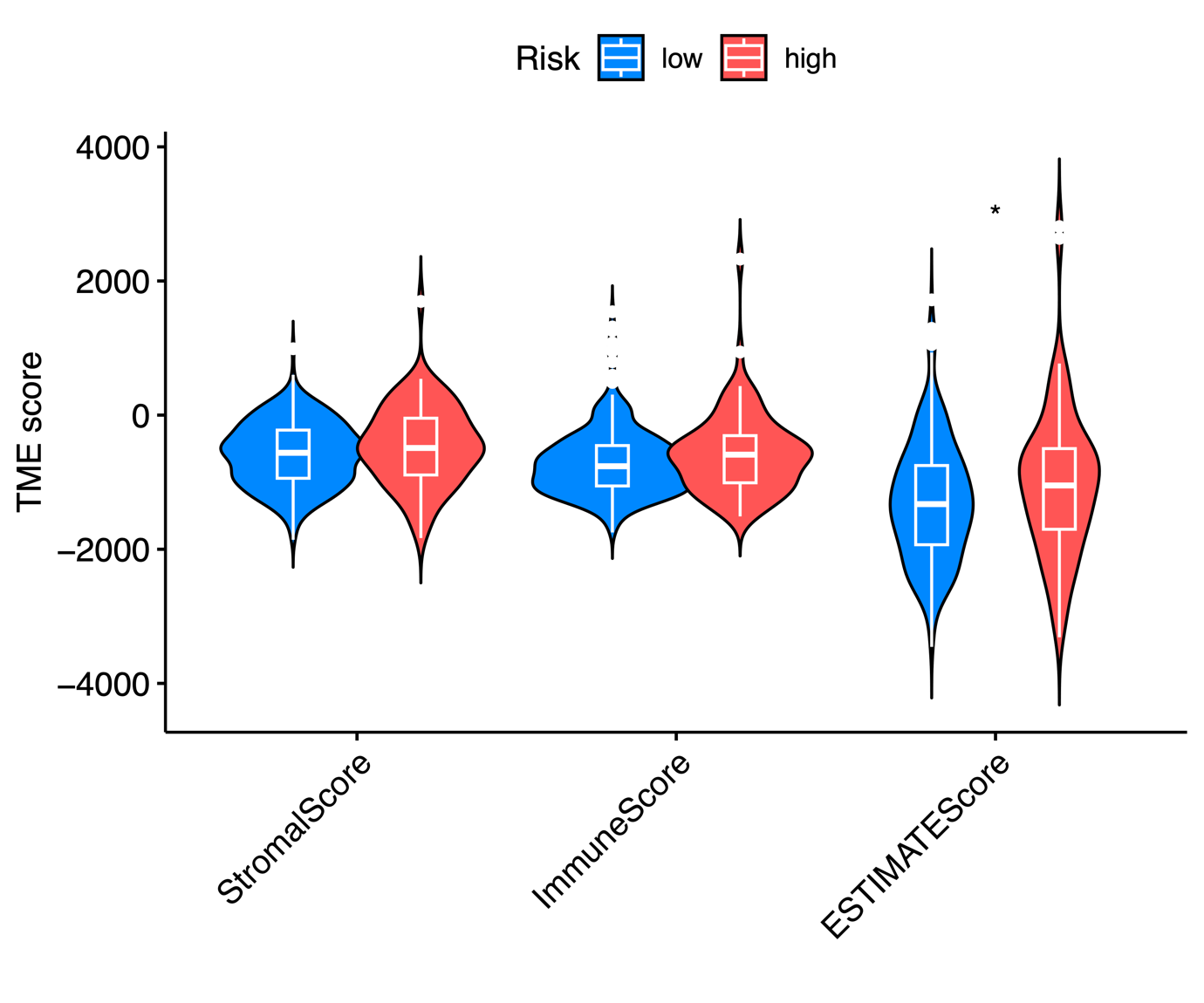


**Supplementary Figure S8: ESTIMATE analysis of stromal, immune, and overall cell scores in different patient risk groups in TCGA-PRAD.**

Violin plots depicting the distribution of StromalScore, ImmuneScore, and ESTIMATEScore between high-risk and low-risk patient groups within the TCGA-PRAD dataset. The scores were calculated using the ESTIMATE algorithm to assess the tumour microenvironment (TME) composition in prostate cancer. The asterisk (*) denotes p < 0.05.


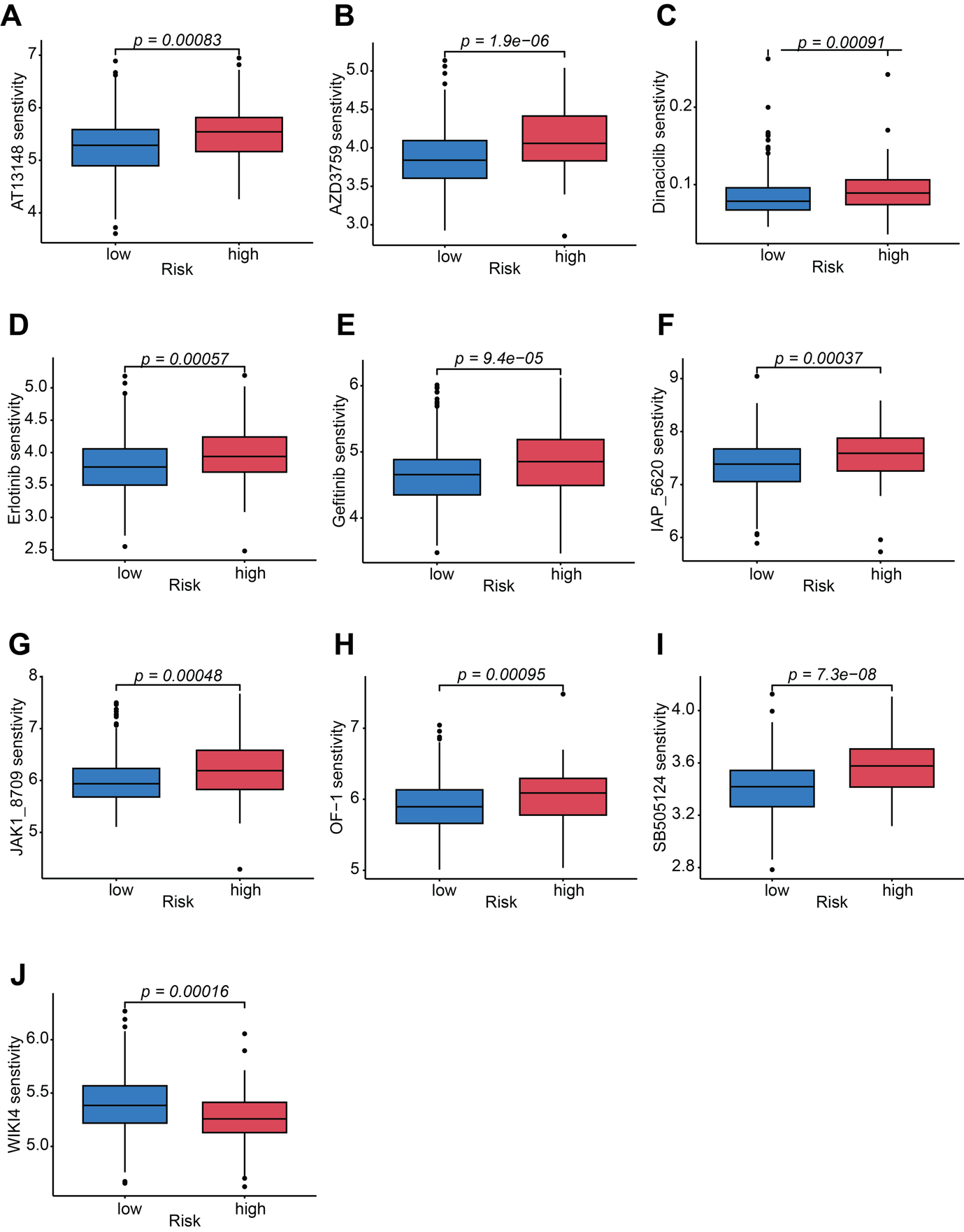


**Supplementary Figure S9: Drug response prediction analysis.**

Box plots illustrating drug response predictions between high- and low-risk patient groups across identified drugs based on the Genomics of Drug Sensitivity in Cancer (GDSC) database.

**Supplementary Tables**

**Supplementary Table S1: Results of meta-analysis.** Gene ID: symbol of the genes considered. Hedges' g: pooled effect size from all studies. Z score: z score derived from pooled effect size and its standard error. P value: p-value calculated from the z score. BH adjusted p: Benjamini-Hochberg.adjusted p-value.

| **Gene ID** | **Hedges' g** | **Z score** | **p value** | **BH adjusted p** |
| --- | --- | --- | --- | --- |
| APEX1 | 0.849653888 | 9.978472625 | 1.89E-23 | 2.78E-22 |
| APOF | 1.299424649 | 14.73687761 | 3.74E-49 | 1.64E-47 |
| BZW2 | 0.815405236 | 3.358503473 | 0.000783657 | 0.001188997 |
| CACNA1D | 0.99878387 | 4.016617589 | 5.90E-05 | 0.000112945 |
| CAMKK2 | 1.15082879 | 5.35005732 | 8.79E-08 | 2.98E-07 |
| DANCR | 1.419689684 | 8.094174658 | 5.77E-16 | 4.23E-15 |
| DDTL | 0.3585882 | 1.53509522 | 0.12476044 | 0.152484983 |
| DPM2 | 0.752573481 | 4.655183971 | 3.24E-06 | 7.50E-06 |
| EEF1D | 0.194644293 | 1.088918968 | 0.276189621 | 0.296398618 |
| ERBB3 | 0.858474851 | 4.706598896 | 2.52E-06 | 6.16E-06 |
| FASN | 0.874862596 | 4.745155632 | 2.08E-06 | 5.39E-06 |
| FBP1 | 0.936286561 | 5.44824211 | 5.09E-08 | 1.87E-07 |
| FKBP4 | 0.492542957 | 2.559715664 | 0.010475783 | 0.013967711 |
| GJB1 | 1.218925407 | 10.76687579 | 4.93E-27 | 1.09E-25 |
| GLT8D2 | -0.383859279 | -1.703926984 | 0.088394729 | 0.114393179 |
| GMDS | 1.086614034 | 9.319585849 | 1.17E-20 | 1.28E-19 |
| HOXC6 | 1.687507243 | 5.166752767 | 2.38E-07 | 6.75E-07 |
| IMPDH2 | 1.264210567 | 7.143207932 | 9.12E-13 | 5.01E-12 |
| LAMTOR2 | 0.63267014 | 3.375114534 | 0.00073785 | 0.001159478 |
| MRPL24 | 0.720672381 | 3.170552155 | 0.001521495 | 0.002231526 |
| MYC | 0.987568473 | 5.468361555 | 4.54E-08 | 1.82E-07 |
| MYL5 | 0.765249519 | 3.7517078 | 0.000175634 | 0.000321996 |
| NOP16 | 0.860697313 | 4.286096194 | 1.82E-05 | 4.00E-05 |
| OR51F2 | 0.708138503 | 2.769228379 | 0.005618923 | 0.00772602 |
| PHB2 | 0.43076462 | 5.161114582 | 2.45E-07 | 6.75E-07 |
| PILRB | 0.279325806 | 1.468223335 | 0.142043568 | 0.168916675 |
| POLD2 | 1.126999661 | 7.575992813 | 3.56E-14 | 2.24E-13 |
| PSMG4 | 0.803972863 | 3.517516664 | 0.000435605 | 0.000709875 |
| PTPRF | -0.175051079 | -0.851189902 | 0.394663871 | 0.403842101 |
| RAB25 | 0.481577898 | 3.137785325 | 0.001702295 | 0.002416161 |
| RPLP0 | 0.626741742 | 4.246071974 | 2.18E-05 | 4.56E-05 |
| SDF4 | 0.234829852 | 1.100354414 | 0.271177732 | 0.296398618 |
| SLC7A11 | 0.875234817 | 3.630260593 | 0.000283135 | 0.000498318 |
| SMIM22 | 0.769126316 | 5.70370172 | 1.17E-08 | 5.73E-08 |
| SMUG1 | 0.542571804 | 4.204525616 | 2.62E-05 | 5.23E-05 |
| TMED3 | 0.719514347 | 8.514427991 | 1.67E-17 | 1.47E-16 |
| TMEM183A | 0.2157998 | 1.445025896 | 0.148450612 | 0.171890182 |
| TMEM97 | 0.535184344 | 3.583577441 | 0.00033892 | 0.000573557 |
| TPI1 | 0.126802208 | 0.750483893 | 0.452963321 | 0.452963321 |
| TRPM4 | 0.928076652 | 1.5820628 | 0.113635235 | 0.142855724 |
| TRPV6 | 0.27444777 | 0.984894626 | 0.324675833 | 0.340136587 |
| TTLL12 | 1.115796268 | 5.254408413 | 1.49E-07 | 4.67E-07 |
| ZNHIT1 | 0.114831164 | 1.150370613 | 0.249991258 | 0.28204142 |
| ZWINT | 0.627890873 | 5.607428716 | 2.05E-08 | 9.04E-08 |

**Supplementary Table S2: Intersection of epithelial cell marker genes and differentially expressed genes in prostate tumour from meta-analysis.** p_val: p-value for the differential expression of the gene. avg_log2FC: average log2 fold change of gene expression between the cluster of interest and all others. pct.1: percentage of cells expressing the gene in the cluster of interest. pct.2: percentage of cells expressing the gene in all other clusters. p_val_adj: Adjusted p-value (FDR) for multiple testing using Benjamini-Hochberg method. cluster: identifier for the cell cluster. gene: symbol of gene considered.

| **p_val** | **avg_log2FC** | **pct.1** | **pct.2** | **p_val_adj** | **cluster** | **gene** |
| --- | --- | --- | --- | --- | --- | --- |
| 0 | 1.295 | 0.477 | 0.039 | 0 | Epithelial_cells | SMIM22 |
| 0 | 0.860 | 0.371 | 0.050 | 0 | Epithelial_cells | FASN |
| 0 | 0.794 | 0.421 | 0.014 | 0 | Epithelial_cells | RAB25 |
| 0 | 0.743 | 0.359 | 0.021 | 0 | Epithelial_cells | ERBB3 |
| 1.80E-289 | 0.976 | 0.326 | 0.039 | 6.05E-285 | Epithelial_cells | CACNA1D |
| 4.90E-274 | 0.819 | 0.504 | 0.145 | 1.64E-269 | Epithelial_cells | TMED3 |
| 3.49E-257 | 0.677 | 0.455 | 0.117 | 1.17E-252 | Epithelial_cells | FBP1 |
| 1.39E-200 | 0.690 | 0.389 | 0.109 | 4.65E-196 | Epithelial_cells | CAMKK2 |
| 3.73E-175 | 0.714 | 0.877 | 0.878 | 1.25E-170 | Epithelial_cells | RPLP0 |
| 8.95E-150 | 0.542 | 0.417 | 0.151 | 3.00E-145 | Epithelial_cells | FKBP4 |
| 7.49E-125 | 0.566 | 0.451 | 0.197 | 2.51E-120 | Epithelial_cells | IMPDH2 |
| 6.79E-98 | 0.425 | 0.347 | 0.139 | 2.28E-93 | Epithelial_cells | POLD2 |
| 1.94E-43 | 0.154 | 0.252 | 0.120 | 6.49E-39 | Epithelial_cells | DPM2 |
| 2.59E-37 | 0.354 | 0.539 | 0.374 | 8.69E-33 | Epithelial_cells | PHB2 |
| 4.59E-25 | 0.144 | 0.257 | 0.150 | 1.54E-20 | Epithelial_cells | PSMG4 |
| 5.98E-17 | 0.205 | 0.471 | 0.340 | 2.01E-12 | Epithelial_cells | APEX1 |
| 9.63E-16 | 0.215 | 0.388 | 0.278 | 3.23E-11 | Epithelial_cells | LAMTOR2 |

**Supplementary Table S3: Results of Kaplan-Meier survival analysis with log-rank test for the 17 epithelial cell marker genes in prostate cancer.** gene: symbol of the gene considered. pvalue: p-value indicating the statistical significance of the survival difference associated with the gene expression levels, calculated using the log-rank test. Only the significant ones are shown.

| **gene** | **pvalue** |
| --- | --- |
| SMIM22 | 0.004930084 |
| FASN | 0.015566028 |
| RAB25 | 0.003381314 |
| ERBB3 | 0.003773425 |
| CACNA1D | 0.034090247 |
| TMED3 | 0.000495356 |
| FBP1 | 0.013521739 |
| FKBP4 | 0.003924431 |
| PHB2 | 0.001274039 |
| PSMG4 | 0.013848811 |
| APEX1 | 0.024106054 |
| LAMTOR2 | 0.005759689 |

**Supplementary Table S4: Construction and validation of epithelial cell marker gene based signatures using machine learning algorithms across TCGA-PRAD, Taylor (GSE21034), Cambridge (GSE70768), CIT (E-MTAB-6128), and DKFZ cohorts.** Model performance was evaluated using the concordance index (C-index) and is displayed per model per cohort. C-index_mean: the average C-index across cohorts for each model; TCGA: The Cancer Genome Atlas; PRAD: prostate adenocarcinoma; RSF: random survival forest; LASSO: least absolute shrinkage and selection operator; GBM: generalised boosted regression modelling; Enet: elastic net; plsRcox: partial least squares regression for Cox; SVM: support vector machine; SuperPC: supervised principal components.

| **Model** | **TCGA** | **Taylor** | **Cambridge** | **CIT** | **DKFZ** | **C-index_mean** |
| --- | --- | --- | --- | --- | --- | --- |
| RSF | 0.964796887 | 0.610981308 | 0.644768856 | 0.739583333 | 0.78533475 | 0.749093027 |
| Lasso + RSF | 0.961878735 | 0.617601246 | 0.648418491 | 0.735416667 | 0.77205101 | 0.74707323 |
| CoxBoost + RSF | 0.961878735 | 0.617601246 | 0.648418491 | 0.735416667 | 0.77205101 | 0.74707323 |
| RSF + GBM | 0.791606837 | 0.558800623 | 0.673965937 | 0.8125 | 0.761424017 | 0.719659483 |
| StepCox[both] + RSF | 0.952707397 | 0.575934579 | 0.610705596 | 0.65625 | 0.778958555 | 0.714911226 |
| StepCox[backward] + RSF | 0.952707397 | 0.575934579 | 0.610705596 | 0.65625 | 0.778958555 | 0.714911226 |
| GBM | 0.813886702 | 0.588395639 | 0.565693431 | 0.770833333 | 0.776301807 | 0.703022182 |
| Lasso + GBM | 0.808143036 | 0.579439252 | 0.566909976 | 0.75625 | 0.75770457 | 0.693689367 |
| CoxBoost + GBM | 0.808143036 | 0.579439252 | 0.566909976 | 0.75625 | 0.75770457 | 0.693689367 |
| StepCox[both] + GBM | 0.799944416 | 0.56347352 | 0.604622871 | 0.710416667 | 0.758235919 | 0.687338679 |
| StepCox[backward] + GBM | 0.799944416 | 0.56347352 | 0.604622871 | 0.710416667 | 0.758235919 | 0.687338679 |
| RSF + survival-SVM | 0.600537311 | 0.606308411 | 0.618004866 | 0.808333333 | 0.713071201 | 0.669251024 |
| RSF + Ridge | 0.634397147 | 0.580607477 | 0.591240876 | 0.74375 | 0.761424017 | 0.662283903 |
| RSF + Enet[α=0.1] | 0.635555144 | 0.580607477 | 0.587591241 | 0.7375 | 0.763549416 | 0.660960655 |
| RSF + Enet[α=0.2] | 0.635786743 | 0.579439252 | 0.586374696 | 0.739583333 | 0.761955367 | 0.660627878 |
| RSF + Enet[α=0.3] | 0.635925703 | 0.57788162 | 0.583941606 | 0.735416667 | 0.762486716 | 0.659130462 |
| RSF + Enet[α=0.4] | 0.636296262 | 0.578660436 | 0.582725061 | 0.735416667 | 0.762486716 | 0.659117028 |
| RSF + Enet[α=0.6] | 0.636342582 | 0.57788162 | 0.581508516 | 0.733333333 | 0.762486716 | 0.658310553 |
| RSF + Enet[α=0.5] | 0.636249942 | 0.577492212 | 0.581508516 | 0.733333333 | 0.762486716 | 0.658214144 |
| RSF + plsRcox | 0.635555144 | 0.579439252 | 0.576642336 | 0.739583333 | 0.759298618 | 0.658103737 |
| RSF + Enet[α=0.7] | 0.636620501 | 0.578271028 | 0.580291971 | 0.733333333 | 0.761955367 | 0.65809444 |
| RSF + Enet[α=0.8] | 0.636481542 | 0.577492212 | 0.580291971 | 0.733333333 | 0.761955367 | 0.657910885 |
| RSF + Enet[α=0.9] | 0.636388902 | 0.577102804 | 0.580291971 | 0.733333333 | 0.761424017 | 0.657708205 |
| RSF + LASSO | 0.636203622 | 0.577102804 | 0.580291971 | 0.733333333 | 0.761424017 | 0.657671149 |
| RSF + CoxBoost | 0.617675668 | 0.573987539 | 0.590024331 | 0.714583333 | 0.764612115 | 0.652176597 |
| Enet[α=0.7] | 0.639029135 | 0.589174455 | 0.46836983 | 0.772916667 | 0.710414453 | 0.635980908 |
| Enet[α=0.9] | 0.638565936 | 0.588395639 | 0.469586375 | 0.775 | 0.707757705 | 0.635861131 |
| Enet[α=0.8] | 0.638704896 | 0.589174455 | 0.469586375 | 0.772916667 | 0.708820404 | 0.635840559 |
| LASSO | 0.638658576 | 0.588395639 | 0.467153285 | 0.775 | 0.709351753 | 0.635711851 |
| CoxBoost | 0.638380657 | 0.587227414 | 0.462287105 | 0.777083333 | 0.710414453 | 0.635078592 |
| Enet[α=0.5] | 0.638843856 | 0.589563863 | 0.46836983 | 0.775 | 0.703506908 | 0.635056891 |
| Enet[α=0.6] | 0.638797536 | 0.589563863 | 0.467153285 | 0.772916667 | 0.706695005 | 0.635025271 |
| Lasso + survival-SVM | 0.623465654 | 0.59423676 | 0.532846715 | 0.789583333 | 0.630712009 | 0.634168894 |
| CoxBoost + survival-SVM | 0.623465654 | 0.59423676 | 0.532846715 | 0.789583333 | 0.630712009 | 0.634168894 |
| Enet[α=0.3] | 0.638519616 | 0.587616822 | 0.47080292 | 0.775 | 0.698193411 | 0.634026554 |
| Enet[α=0.4] | 0.639214415 | 0.588785047 | 0.469586375 | 0.770833333 | 0.701381509 | 0.633960136 |
| CoxBoost + LASSO | 0.639446014 | 0.590732087 | 0.459854015 | 0.775 | 0.704038257 | 0.633814075 |
| CoxBoost + Enet[α=0.7] | 0.639723933 | 0.589563863 | 0.46107056 | 0.775 | 0.702975558 | 0.633666783 |
| Enet[α=0.2] | 0.638473297 | 0.586448598 | 0.474452555 | 0.775 | 0.693411265 | 0.633557143 |
| CoxBoost + Enet[α=0.8] | 0.639353374 | 0.589953271 | 0.459854015 | 0.775 | 0.703506908 | 0.633533514 |
| Lasso + CoxBoost | 0.639260735 | 0.589563863 | 0.45863747 | 0.775 | 0.704038257 | 0.633300065 |
| CoxBoost + Enet[α=0.6] | 0.639307055 | 0.589174455 | 0.46107056 | 0.775 | 0.701912859 | 0.633292986 |
| CoxBoost + Enet[α=0.9] | 0.639260735 | 0.590732087 | 0.459854015 | 0.772916667 | 0.703506908 | 0.633254082 |
| Enet[α=0.1] | 0.637500579 | 0.586448598 | 0.48053528 | 0.777083333 | 0.684378321 | 0.633189222 |
| CoxBoost + Enet[α=0.5] | 0.639307055 | 0.589563863 | 0.462287105 | 0.772916667 | 0.701381509 | 0.63309124 |
| CoxBoost + Enet[α=0.2] | 0.638519616 | 0.589953271 | 0.46836983 | 0.775 | 0.692879915 | 0.632944526 |
| CoxBoost + Enet[α=0.3] | 0.639584974 | 0.588785047 | 0.46593674 | 0.770833333 | 0.698193411 | 0.632666701 |
| CoxBoost + Enet[α=0.1] | 0.638149057 | 0.589174455 | 0.472019465 | 0.772916667 | 0.689160468 | 0.632284022 |
| Lasso + plsRcox | 0.638519616 | 0.590342679 | 0.45863747 | 0.770833333 | 0.702975558 | 0.632261731 |
| CoxBoost + plsRcox | 0.638519616 | 0.590342679 | 0.45863747 | 0.770833333 | 0.702975558 | 0.632261731 |
| Lasso + StepCox[both] | 0.620037982 | 0.570482866 | 0.553527981 | 0.729166667 | 0.687566419 | 0.632156383 |
| Lasso + StepCox[backward] | 0.620037982 | 0.570482866 | 0.553527981 | 0.729166667 | 0.687566419 | 0.632156383 |
| StepCox[both] | 0.620037982 | 0.570482866 | 0.553527981 | 0.729166667 | 0.687566419 | 0.632156383 |
| StepCox[backward] | 0.620037982 | 0.570482866 | 0.553527981 | 0.729166667 | 0.687566419 | 0.632156383 |
| CoxBoost + StepCox[both] | 0.620037982 | 0.570482866 | 0.553527981 | 0.729166667 | 0.687566419 | 0.632156383 |
| CoxBoost + StepCox[backward] | 0.620037982 | 0.570482866 | 0.553527981 | 0.729166667 | 0.687566419 | 0.632156383 |
| CoxBoost + Enet[α=0.4] | 0.639538654 | 0.588006231 | 0.462287105 | 0.76875 | 0.69978746 | 0.63167389 |
| CoxBoost + Ridge | 0.636666821 | 0.588006231 | 0.479318735 | 0.775 | 0.673219979 | 0.630442353 |
| plsRcox | 0.638519616 | 0.593847352 | 0.459854015 | 0.76875 | 0.690223167 | 0.63023883 |
| Ridge | 0.63713002 | 0.584890966 | 0.479318735 | 0.764583333 | 0.663124336 | 0.625809478 |
| survival-SVM | 0.625781648 | 0.60046729 | 0.50486618 | 0.775 | 0.61477152 | 0.624177327 |
| RSF + StepCox[both] | 0.61299736 | 0.532320872 | 0.520681265 | 0.697916667 | 0.733262487 | 0.61943573 |
| RSF + StepCox[backward] | 0.61299736 | 0.532320872 | 0.520681265 | 0.697916667 | 0.733262487 | 0.61943573 |
| StepCox[both] + Enet[α=0.1] | 0.620501181 | 0.572040498 | 0.445255474 | 0.73125 | 0.687566419 | 0.611322715 |
| StepCox[backward] + Enet[α=0.1] | 0.620501181 | 0.572040498 | 0.445255474 | 0.73125 | 0.687566419 | 0.611322715 |
| StepCox[both] + Enet[α=0.8] | 0.620640141 | 0.570872274 | 0.446472019 | 0.73125 | 0.686503719 | 0.611147631 |
| StepCox[both] + Enet[α=0.9] | 0.620640141 | 0.570872274 | 0.446472019 | 0.73125 | 0.686503719 | 0.611147631 |
| StepCox[backward] + Enet[α=0.8] | 0.620640141 | 0.570872274 | 0.446472019 | 0.73125 | 0.686503719 | 0.611147631 |
| StepCox[backward] + Enet[α=0.9] | 0.620640141 | 0.570872274 | 0.446472019 | 0.73125 | 0.686503719 | 0.611147631 |
| StepCox[both] + LASSO | 0.620501181 | 0.570482866 | 0.446472019 | 0.73125 | 0.686503719 | 0.611041957 |
| StepCox[backward] + LASSO | 0.620501181 | 0.570482866 | 0.446472019 | 0.73125 | 0.686503719 | 0.611041957 |
| StepCox[both] + plsRcox | 0.620176942 | 0.570093458 | 0.446472019 | 0.73125 | 0.687035069 | 0.611005498 |
| StepCox[backward] + plsRcox | 0.620176942 | 0.570093458 | 0.446472019 | 0.73125 | 0.687035069 | 0.611005498 |
| StepCox[both] + Enet[α=0.7] | 0.620640141 | 0.570482866 | 0.446472019 | 0.73125 | 0.68597237 | 0.610963479 |
| StepCox[backward] + Enet[α=0.7] | 0.620640141 | 0.570482866 | 0.446472019 | 0.73125 | 0.68597237 | 0.610963479 |
| StepCox[both] + Ridge | 0.619343184 | 0.574376947 | 0.441605839 | 0.735416667 | 0.683846971 | 0.610917922 |
| StepCox[backward] + Ridge | 0.619343184 | 0.574376947 | 0.441605839 | 0.735416667 | 0.683846971 | 0.610917922 |
| StepCox[both] + Enet[α=0.2] | 0.62087174 | 0.570482866 | 0.446472019 | 0.729166667 | 0.687035069 | 0.610805672 |
| StepCox[backward] + Enet[α=0.2] | 0.62087174 | 0.570482866 | 0.446472019 | 0.729166667 | 0.687035069 | 0.610805672 |
| StepCox[both] + CoxBoost | 0.620593821 | 0.570872274 | 0.446472019 | 0.729166667 | 0.68597237 | 0.61061543 |
| StepCox[backward] + CoxBoost | 0.620593821 | 0.570872274 | 0.446472019 | 0.729166667 | 0.68597237 | 0.61061543 |
| StepCox[both] + Enet[α=0.3] | 0.620640141 | 0.571261682 | 0.445255474 | 0.729166667 | 0.686503719 | 0.610565537 |
| StepCox[backward] + Enet[α=0.3] | 0.620640141 | 0.571261682 | 0.445255474 | 0.729166667 | 0.686503719 | 0.610565537 |
| StepCox[both] + Enet[α=0.5] | 0.620362222 | 0.571261682 | 0.445255474 | 0.729166667 | 0.686503719 | 0.610509953 |
| StepCox[backward] + Enet[α=0.5] | 0.620362222 | 0.571261682 | 0.445255474 | 0.729166667 | 0.686503719 | 0.610509953 |
| StepCox[both] + Enet[α=0.4] | 0.620501181 | 0.570872274 | 0.445255474 | 0.729166667 | 0.686503719 | 0.610459863 |
| StepCox[backward] + Enet[α=0.4] | 0.620501181 | 0.570872274 | 0.445255474 | 0.729166667 | 0.686503719 | 0.610459863 |
| StepCox[both] + Enet[α=0.6] | 0.620547501 | 0.570482866 | 0.445255474 | 0.729166667 | 0.686503719 | 0.610391246 |
| StepCox[backward] + Enet[α=0.6] | 0.620547501 | 0.570482866 | 0.445255474 | 0.729166667 | 0.686503719 | 0.610391246 |
| StepCox[both] + survival-SVM | 0.587104544 | 0.56970405 | 0.48783455 | 0.777083333 | 0.595642933 | 0.603473882 |
| StepCox[backward] + survival-SVM | 0.587104544 | 0.56970405 | 0.48783455 | 0.777083333 | 0.595642933 | 0.603473882 |
| StepCox[both] + SuperPC | 0.509796656 | 0.537383178 | 0.547445255 | 0.54375 | 0.604675877 | 0.548610193 |
| StepCox[backward] + SuperPC | 0.509796656 | 0.537383178 | 0.547445255 | 0.54375 | 0.604675877 | 0.548610193 |
| RSF + SuperPC | 0.485756635 | 0.521028037 | 0.53163017 | 0.6375 | 0.562699256 | 0.54772282 |
| Lasso + SuperPC | 0.536893789 | 0.565809969 | 0.553527981 | 0.529166667 | 0.540382572 | 0.545156195 |
| CoxBoost + SuperPC | 0.536893789 | 0.565809969 | 0.553527981 | 0.529166667 | 0.540382572 | 0.545156195 |
| SuperPC | 0.500625318 | 0.515965732 | 0.52919708 | 0.60625 | 0.498937301 | 0.530195086 |

**Supplementary Table S5: Overview of 11 signature genes and their associations with prostate cancer.**

| **Gene** | **Full name** | **Function in prostate cancer** |
| --- | --- | --- |
| LAMTOR2 | Late Endosomal/Lysosomal Adaptor, MAPK and MTOR Activator 2 | Although other members of the LAMTOR complex have been associated prostate cancer [1], and LAMTOR2 itself has been associated with other cancers such as uveal melanoma [2] and pancreatic ductal carcinoma [3], no specific relationship of LAMTOR2 with prostate cancer has been documented. |
| RAB25 | RAB25, Member RAS Oncogene Family | RAB25 plays an oncogenic role in prostate cancer, with elevated expression linked to disease progression and increased risk of biochemical recurrence following radical prostatectomy. Suppression of RAB25 reduces proliferation, migration, and invasion in prostate cancer cells (LNCaP) [4].  RAB25 has been showed to become increasingly active as prostate cancer progresses, from less aggressive cell lines (Pr117) to highly aggressive metastatic lines (Pr14C1). It is associated with tumorigenicity and aggressiveness and may serve as a biomarker or therapeutic target [5]. |
| FBP1 | Fructose-Bisphosphatase 1 | Knockdown of FBP1 expression accelerates prostate tumour growth and contributes to resistance against anti-PD-L1 immunotherapy in preclinical mouse models [6]. |
| TMED3 | Transmembrane P24 Trafficking Protein 3 | TMED3 has been identified as an epithelial cell marker and a prognostic biomarker for biochemical recurrence-free survival in prostate cancer patients. It is upregulated in prostate tumour tissues (e.g., VCaP and LNCaP cell lines) and promotes malignant proliferation of prostate cancer cells, suggesting its potential as a therapeutic target [7]. |
| FASN | Fatty Acid Synthase | FASN is significantly overexpressed in prostate cancer and is under investigation as a therapeutic target in ongoing clinical trials. Research has shown that FASN upregulation plays a critical role in prostatic tumorigenesis and is elevated in metastatic prostate cancer [8,9].  Targeting FASN with pharmacological inhibitors has demonstrated efficacy in reducing tumour growth in preclinical models of castration-resistant prostate cancer (CRPC) by disrupting androgen receptor (AR) signalling pathways. The FASN inhibitor IPI-9119 has been shown to induce apoptosis in prostate cancer cells [10,11]. |
| PHB2 | Prohibitin 2 | Upregulation of PHB2 has been reported to inhibit androgen receptor (AR) activity and prostate cancer cell proliferation and migration [12]. |
| FKBP4 | FKBP Prolyl Isomerase 4 | FKBP4 is a potential therapeutic target in CRPC [13]. Its upregulation is associated with worse survival in hormone-naïve prostate cancer patients and may serve as a promising biomarker for predicting androgen deprivation therapy (ADT) response [14]. |
| ERBB3 | Erb-B2 Receptor Tyrosine Kinase 3 | Overexpression of ERBB3 in prostate cancer is associated with quicker progression to castration resistance and shorter survival in advanced cases [15]. It has been implicated in resistance to enzalutamide and may help stratify patients for intensified therapy, such as targeting ERBB3 to improve sensitivity to androgen receptor (AR)-targeted therapies [16]. |
| PSMG4 | Proteasome Assembly Chaperone 4 | Although PSMG4 has previously been associated with lung adenocarcinoma [17], no direct association with prostate cancer has been reported to date |
| CACNA1D | Calcium Voltage-Gated Channel Subunit Alpha1 D | CACNA1D was reported to be highly expressed at both mRNA and protein levels in prostate cancer and is associated with castration-resistant and aggressive phenotypes. It has been demonstrated to play a role in calcium signalling pathways that support tumour proliferation and survival under androgen-deprivation therapy [18,19]. |
| APEX1 | Apurinic/Apyrimidinic Endodeoxyribonuclease 1 | APEX1 overexpression has been identified as an independent prognostic marker in ERG-negative prostate cancers [20].  APEX1 and survivin have been showed to be significantly overexpressed in prostate cancer compared to noncancerous controls. Targeting APEX1 with redox-specific small-molecule inhibitors, such as APX3330 and APX2009, can suppress prostate cancer cell proliferation and induces cell cycle arrest [21]. |

**Supplementary Table S6: Results of Kaplan-Meier survival analysis with log-rank test on the panel genes in GSE53922.** gene: symbol of the gene considered. pvalue: p-value indicating the statistical significance of the survival difference associated with the gene expression levels, calculated using the log-rank test.

| **gene** | **pvalue** |
| --- | --- |
| RAB25 | 8.24E-05 |
| POLD2 | 0.000275732 |
| APEX1 | 0.00032247 |
| DPM2 | 0.000746746 |
| RPLP0 | 0.000883372 |
| IMPDH2 | 0.002085736 |
| ERBB3 | 0.011075408 |
| CAMKK2 | 0.011540255 |
| FBP1 | 0.016484092 |
| CACNA1D | 0.022021119 |
| PHB2 | 0.040083781 |
| LAMTOR2 | 0.072494181 |
| FASN | 0.169199852 |
| LOC389362 | 0.174039038 |
| FKBP4 | 0.175532036 |
| TMED3 | 0.353777683 |

**References**

1. Gamallat Y, Alwazan H, Turko R, et al. Elevated LAMTOR4 Expression Is Associated with Lethal Prostate Cancer and Its Knockdown Decreases Cell Proliferation, Invasion, and Migration In Vitro. *Int J Mol Sci*. Jul 25 2024;25(15)doi:10.3390/ijms25158100

2. Wang J, Liu M, Sun J, Zhang Z. Immunogenic profiling of metastatic uveal melanoma discerns a potential signature related to prognosis. *J Cancer Res Clin Oncol*. Jan 21 2024;150(1):23. doi:10.1007/s00432-023-05542-z

3. Xu J, Quan G, Huang W, Jiang J. VSIG2 promotes malignant progression of pancreatic ductal adenocarcinoma by enhancing LAMTOR2-mediated mTOR activation. *Cell Commun Signal*. Aug 25 2023;21(1):223. doi:10.1186/s12964-023-01209-x

4. Hu C, Chen B, Zhou Y, Shan Y. High expression of Rab25 contributes to malignant phenotypes and biochemical recurrence in patients with prostate cancer after radical prostatectomy. *Cancer Cell Int*. 2017;17:45. doi:10.1186/s12935-017-0411-0

5. Calvo A, Xiao N, Kang J, et al. Alterations in gene expression profiles during prostate cancer progression: functional correlations to tumorigenicity and down-regulation of selenoprotein-P in mouse and human tumors. *Cancer Res*. Sep 15 2002;62(18):5325-35.

6. Wang B, Zhou Y, Zhang J, Jin X, Wu H, Huang H. Fructose-1,6-bisphosphatase loss modulates STAT3-dependent expression of PD-L1 and cancer immunity. *Theranostics*. 2020;10(3):1033-1045. doi:10.7150/thno.38137

7. Zhu W, Zeng H, Huang J, et al. Integrated machine learning identifies epithelial cell marker genes for improving outcomes and immunotherapy in prostate cancer. *J Transl Med*. Nov 4 2023;21(1):782. doi:10.1186/s12967-023-04633-2

8. Shurbaji MS, Kalbfleisch JH, Thurmond TS. Immunohistochemical detection of a fatty acid synthase (OA-519) as a predictor of progression of prostate cancer. *Hum Pathol*. Sep 1996;27(9):917-21. doi:10.1016/s0046-8177(96)90218-x

9. Rossi S, Graner E, Febbo P, et al. Fatty acid synthase expression defines distinct molecular signatures in prostate cancer. *Mol Cancer Res*. Aug 2003;1(10):707-15.

10. Zadra G, Ribeiro CF, Chetta P, et al. Inhibition of de novo lipogenesis targets androgen receptor signaling in castration-resistant prostate cancer. *Proc Natl Acad Sci U S A*. Jan 8 2019;116(2):631-640. doi:10.1073/pnas.1808834116

11. Falchook G, Infante J, Arkenau HT, et al. First-in-human study of the safety, pharmacokinetics, and pharmacodynamics of first-in-class fatty acid synthase inhibitor TVB-2640 alone and with a taxane in advanced tumors. *EClinicalMedicine*. Apr 2021;34:100797. doi:10.1016/j.eclinm.2021.100797

12. Zhang Y, Wang LN, Lin YN, et al. The novel long noncoding RNA LOC283070 is involved in the transition of LNCaP cells into androgen-independent cells via its interaction with PHB2. *Asian J Androl*. Sep-Oct 2018;20(5):511-517. doi:10.4103/aja.aja_36_18

13. De Leon JT, Iwai A, Feau C, et al. Targeting the regulation of androgen receptor signaling by the heat shock protein 90 cochaperone FKBP52 in prostate cancer cells. *Proc Natl Acad Sci U S A*. Jul 19 2011;108(29):11878-83. doi:10.1073/pnas.1105160108

14. Federer-Gsponer JR, Quintavalle C, Müller DC, et al. Delineation of human prostate cancer evolution identifies chromothripsis as a polyclonal event and FKBP4 as a potential driver of castration resistance. *J Pathol*. May 2018;245(1):74-84. doi:10.1002/path.5052

15. Gil V, Miranda S, Riisnaes R, et al. HER3 Is an Actionable Target in Advanced Prostate Cancer. *Cancer Res*. Dec 15 2021;81(24):6207-6218. doi:10.1158/0008-5472.Can-21-3360

16. Vellky JE, Kirkpatrick BJ, Gutgesell LC, et al. ERBB3 Overexpression is Enriched in Diverse Patient Populations with Castration-sensitive Prostate Cancer and is Associated with a Unique AR Activity Signature. *Clin Cancer Res*. Apr 15 2024;30(8):1530-1543. doi:10.1158/1078-0432.Ccr-23-2161

17. Xuan DTM, Yeh IJ, Su CY, et al. Prognostic and Immune Infiltration Value of Proteasome Assembly Chaperone (PSMG) Family Genes in Lung Adenocarcinoma. *Int J Med Sci*. 2023;20(1):87-101. doi:10.7150/ijms.78590

18. Chen R, Zeng X, Zhang R, et al. Cav1.3 channel α1D protein is overexpressed and modulates androgen receptor transactivation in prostate cancers. *Urol Oncol*. Jul 2014;32(5):524-36. doi:10.1016/j.urolonc.2013.05.011

19. O'Reilly D, Downing T, Kouba S, et al. CaV1.3 enhanced store operated calcium promotes resistance to androgen deprivation in prostate cancer. *Cell Calcium*. May 2022;103:102554. doi:10.1016/j.ceca.2022.102554

20. Juhnke M, Heumann A, Chirico V, et al. Apurinic/apyrimidinic endonuclease 1 (APE1/Ref-1) overexpression is an independent prognostic marker in prostate cancer without TMPRSS2:ERG fusion. *Mol Carcinog*. Sep 2017;56(9):2135-2145. doi:10.1002/mc.22670

21. McIlwain DW, Fishel ML, Boos A, Kelley MR, Jerde TJ. APE1/Ref-1 redox-specific inhibition decreases survivin protein levels and induces cell cycle arrest in prostate cancer cells. *Oncotarget*. Feb 16 2018;9(13):10962-10977. doi:10.18632/oncotarget.23493
